# S-Nitroso-Proteome in Poplar Leaves in Response to Acute Ozone Stress

**DOI:** 10.1371/journal.pone.0106886

**Published:** 2014-09-05

**Authors:** Elisa Vanzo, Andrea Ghirardo, Juliane Merl-Pham, Christian Lindermayr, Werner Heller, Stefanie M. Hauck, Jörg Durner, Jörg-Peter Schnitzler

**Affiliations:** 1 Research Unit Environmental Simulation, Institute for Biochemical Plant Pathology, Helmholtz Zentrum München, Neuherberg, Germany; 2 Research Unit Protein Science, Helmholtz Zentrum München, Neuherberg, Germany; 3 Institute for Biochemical Plant Pathology, Helmholtz Zentrum München, Neuherberg, Germany; National Taiwan University, Taiwan

## Abstract

Protein S-nitrosylation, the covalent binding of nitric oxide (NO) to protein cysteine residues, is one of the main mechanisms of NO signaling in plant and animal cells. Using a combination of the biotin switch assay and label-free LC-MS/MS analysis, we revealed the S-nitroso-proteome of the woody model plant *Populus* x *canescens*. Under normal conditions, constitutively S-nitrosylated proteins in poplar leaves and calli comprise all aspects of primary and secondary metabolism. Acute ozone fumigation was applied to elicit ROS-mediated changes of the S-nitroso-proteome. This treatment changed the total nitrite and nitrosothiol contents of poplar leaves and affected the homeostasis of 32 S-nitrosylated proteins. Multivariate data analysis revealed that ozone exposure negatively affected the S-nitrosylation status of leaf proteins: 23 proteins were de-nitrosylated and 9 proteins had increased S-nitrosylation content compared to the control. Phenylalanine ammonia-lyase 2 (log2[ozone/control] = −3.6) and caffeic acid O-methyltransferase (−3.4), key enzymes catalyzing important steps in the phenylpropanoid and subsequent lignin biosynthetic pathways, respectively, were de-nitrosylated upon ozone stress. Measuring the *in vivo* and *in vitro* phenylalanine ammonia-lyase activity indicated that the increase of the phenylalanine ammonia-lyase activity in response to acute ozone is partly regulated by de-nitrosylation, which might favor a higher metabolic flux through the phenylpropanoid pathway within minutes after ozone exposure.

## Introduction

Nitric oxide (NO), a volatile nitrogen compound, is widely recognized as a signaling molecule in various organisms. In plants, NO is involved in the regulation of various developmental processes, such as root growth, seed germination, flowering, pollen tube re-orientation and stomatal closure [1]–[6]. Furthermore, NO plays important roles in plant responses to biotic and abiotic stresses [7], [8].

Different modes of NO signaling have been reported to mediate this abundance of function in plants. Most NO signaling is accomplished through the posttranslational modification (PTM) of target proteins, such as (i) the nitration of protein tyrosine moieties, (ii) binding to metal centers or (iii) the nitrosylation of cysteine residues [9]. S-nitrosylation, the reversible attachment of a NO moiety to thiol groups of selected cysteine residues functions as the most important PTM in the context of NO signaling. S-nitrosylation can impact protein functionality, stability and cellular localization [10]. The S-nitrosylation of enzymes regulates their activity either negatively or positively. Several detailed analyses of the S-nitrosylation of specific proteins have used NO donors (i.e., S-nitrosoglutathione (GSNO)) to promote S-nitrosylation *in vitro*. In most cases, the NO donor treatment seems to reduce the enzyme activity in plants [11]–[15]. Reports showing that the protein S-nitrosylation of specific cysteine residues promotes enzyme activity are scarce in plant science, and only a few hints regarding an activity-enhancing effect have been reported for animals [16]–[18]. Although de-nitrosylation represents a less-described aspect of NO signaling, the process of de-nitrosylation is also a strictly regulated event involving two recently proposed enzyme systems: (i) the thioredoxin system, which comprises thioredoxin, thioredoxin reductase and NADPH, and (ii) the glutathione/GSNO reductase system [19]. Some proteins are constitutively S-nitrosylated, and de-nitrosylation has been observed after stimulation, leading to the activation of enzyme activity or *vice versa* [20], [21]. S-nitrosylation and de-nitrosylation together generate the S-nitroso-proteome of a cell, a dynamic and rapidly changing regulatory network, especially under stress conditions.

More than a decade ago, Jaffrey and colleagues introduced the biotin switch assay [22], which facilitates the identification of S-nitrosylated proteins. By utilizing both the biotin switch assay and mass spectrometry (MS), hundreds of putative S-nitrosylation targets have been identified. In pioneering studies in plants, S-nitrosylation was enforced using NO donors on protein extracts [11], [23], [24]. More recent research has focused on identifying endogenously S-nitrosylated proteins in unstressed plants [25] and S-nitrosylation patterns in plants that are exposed to different stresses [15], [26]–[28]. Comparative analysis of the S-nitroso-proteome under control and stress conditions is an important tool to provide information about the biological relevance of NO signaling upon various stress conditions. To date, no information is available regarding ozone-induced changes in the S-nitroso-proteome of plants. Although several studies describe the impact of acute ozone exposure on total proteomes of rice, soybean, wheat, and poplar [29]–[32], the issue of redox-linked protein modifications upon ozone has only been examined in two studies [33], [34]. Ozone exerts bi-functional effects on earth. While stratospheric ozone protects life from harmful ultraviolet radiation, tropospheric ozone is an air pollutant that can induce oxidative stress and cell death in plants [35], [36] causing considerable agricultural crop losses and damage in forest trees [37], [38]. Temporary exposure to ozone at a high-level, termed acute exposure induces changes in gene expression and protein activities often within minutes after the onset of the fumigation [39]. It causes the formation of various reactive oxygen species (ROS) in plant tissues, mainly superoxide anion, hydrogen peroxide (H_2_O_2_) and hydroxyl radicals, which can induce cell-death lesions in ozone-sensitive plants. This rapid accumulation of ROS upon acute ozone treatment resembles the oxidative burst after plant-pathogen interactions [40], [41]. Concomitant with the oxidative burst upon acute ozone fumigation, an accumulation of NO is observed [42]–[44]. It is thought that, in response to ozone, NO and ROS work synergistically to promote a defense response in plants, mimicking the hypersensitive response (HR) that occurs as a result of incompatible plant-pathogen interactions [41], [45]. Therefore, the fine-tuning of the NO/ROS balance is needed [46].

Poplar is the model system for woody plants as its relatively small genome was the first to be sequenced [47]. Also, poplar is suitable for genetic transformation and can be propagated vegetatively, facilitating large-scale production of clones [48]. As demand for renewable bioenergy is increasing, poplar, a fast-growing pioneer tree, is receiving great amounts of attention due to its suitability for heat and power generation [49], [50]. This demand implies more future scientific and economical interest in using poplar for analyzing stress responses in woody plant species. Moreover, while knowledge of S-nitrosylated proteins in herbaceous model plants is broad [11], [26], [28], there is limited information regarding woody plants [51].

In the present study we aimed to identify S-nitrosylated proteins in grey poplar (*Populus* x *canescens*) to improve understanding of the initial steps in plants’ ozone response. By performing a biotin switch assay in conjunction with quantitative (label-free) LC-MS/MS analysis, we tried to answer the following questions: Firstly, are there proteins that are constitutively S-nitrosylated in the green tissue (callus and leaf) of poplar? Secondly, does acute oxidative stress (ozone fumigation) induce quantitative and/or qualitative alterations in the pattern of S-nitrosylated proteins in the leaves? And finally, if there are changes upon oxidative stress, can we deduce a regulation scheme that may explain the physiological relevance of S-nitrosylation signaling during plant ozone response?

Here we report a list of constitutively S-nitrosylated proteins in grey poplar under unstressed conditions. The list comprises many proteins not reported in the context of S-nitrosylation so far. Quantitative (label-free) analysis revealed significant and rapid changes in the S-nitroso-proteome of poplar undergoing acute ozone exposure.

## Materials and Methods

### Plant material, growth conditions and ozone treatment

For the generation of callus tissue, leaf and stem explants of grey poplar (*Populus* x *canescens* INRA clone 7171-B4; syn. *Populus tremula* x *Populus alba* (Aiton.) Smith) were cultured in the dark on callus induction medium for three weeks at 20°C [52]. Explants were transferred to shoot induction medium and were maintained for up to ten weeks under moderate light (16/8 h photoperiod at 125 µmol photons m^−2 ^s^−1^). Fully developed calli (Figure S1) were frozen at −80°C. All media are described elsewhere [53].

The ozone experiments were performed in three independent runs with *P.* x *canescens* plants. Poplar plants were multiplied by micropropagation on half-concentrated MS medium as described elsewhere [53]. After 8 weeks rooted shoots were transferred to soil substrate (50% v/v Fruhstorfer Einheitserde, 50% v/v silica sand (particle size 1–3 mm)) and grown under a plastic lid to maintain high humidity, to which the plants got used, during sterile culture. Plantlets were grown under the following conditions: 27°C/24°C (day/night) and a photoperiod of 16 h with approximately 100 µmol photons m^−2 ^s^−1^ during the light period. Plantlets were adapted to ambient humidity conditions by carefully opening the lid after two weeks. After acclimatization, the plants were planted into 2.2 l pots (25% v/v Fruhstorfer Einheitserde, 25% v/v silica sand (particle size 1–3 mm), 50% v/v perlite) and were transferred to the greenhouse. Before ozone fumigation, the plants were grown in the greenhouse for eight weeks in May and June 2012 until they had attained a height of 60–70 cm and had produced 20–22 leaves. No supplemental lighting was provided. Fertilization was performed with Triabon (Compo, Münster, Germany) and Osmocote (Scotts Miracle-Gro, Marysville, USA) (1∶1, v/v; 10 g per liter of soil).

Plant responses to ozone are closely linked to the effective dose taken up by the plant via the stomata [54]. Thus, flux-based indices that take into account ozone deposition into the leaf are considered a more reliable indicator of potential ozone damage than exposure time and air ozone concentration [55], [56]. To determine the ozone uptake in grey poplar leaves by a given ozone concentration, we quantified the cumulative ozone dose in our experiments. Poplar plants were enclosed in a cuvette made of glass and Teflon (170 l volume, PPFD 250 µmol m^−2 ^s^−1^, air temperature 25°C±1°C, and flux 11.5 l min^−1^). A fan ensured homogeneous mixing of the chamber air to remove boundary layer resistance at the plant surfaces. CO_2_ assimilation, transpiration and foliar ozone flux (nmol m^−2 ^s^−1^) were monitored as differences between cuvette inlet and outlet by infrared-absorption (Fischer-Rosemount Binos 100 4P, Hasselroth, Germany), and chemoluminescence (O341 M, Ansyco Karlsruhe, Germany), respectively (Figure S2). When net CO_2_ assimilation was stable, ozone fumigation (800 ppb) was applied for 1 hour. Ozone destruction on inner surfaces of the cuvette and non-stomatal adsorption on outer plant surfaces were taken into account by measuring empty cuvettes (including covered pots with soil) or darkening the plant in the cuvette, respectively. Application of 800 ppb ozone in the inlet air resulted finally in a cumulative uptake of ozone of 110±19 µmol m^−2^ (n = 6±SE). This ozone ‘dose’ is in the same range as previously described in the context of acute ozone treatments on plants (130–200 µmol ozone m^−2^ [57] and approx. 370 µmol ozone m^−2^ [58]).

Before fumigation started, all plants (control (C) and ozone (O) plants) were allowed to acclimatize to the cuvette conditions for 2 hours (PPFD 250 µmol m^−2 ^s^−1^, air temperature 25°C±1°C). O plants were then exposed to 800 ppb ozone for 1 hour, while control plants (C) were further kept in the control cuvette under ambient air (ozone-free) for 1 hour. For proteomics and biochemical analysis, fully developed leaves (number 9 and 10 from the apex; n = 3 plants for C and O, each) were frozen in liquid nitrogen immediately after exposure. C and O samples were subjected to the biotin switch assay (for detection of S-nitrosylated proteins), overall proteomic analysis (for normalization of S-nitrosylated protein abundance) and *in vivo* PAL activity measurement. Measurements of the *in vitro* phenylalanine ammonia-lyase (PAL) activity were performed on samples taken one month later from grey poplar plants that were grown in the greenhouse for 12 weeks in June, July and August 2012 (leaf number 9 and 10 from the apex).

### Detection of endogenously S-nitrosylated proteins in poplar green tissue by modified biotin switch assay

The detection of *in vivo* S-nitrosylated proteins was performed *via* a modified biotin switch assay [22]. One of the most important modifications was the extraction of proteins in the presence of N-ethylmaleimide (NEM), which ‘freezes’ the S-nitrosylation pattern present at the moment of extraction by blocking all accessible thiol residues. A second change concerned the reduction step. Ascorbate, which serves as specific cysteine-NO reducing agent, was replaced by sinapic acid (SIN) due to its greater degree of specificity [59], [60]. In brief, frozen leaf powder (either callus tissue or leaf tissue from control or ozone-treated plants) was mixed with HENT buffer (100 mM HEPES-NaOH pH 7.4, 10 mM EDTA, 0.1 mM Neocuproine, 1% (v/v) Triton X-100) in a mixing ratio of leaf powder:buffer 1∶5 (w/v). The HENT buffer contained 30 mM NEM and protease inhibitor cocktail tablets (Complete, Roche, Grenzach-Wyhlen, Germany). The homogenate was mixed on a shaker for 30 s, incubated on ice for 15 min and centrifuged twice (14 000 *g* for 10 min). The protein concentration of the supernatant was adjusted to 1 µg/*µ*l using the HENT buffer. For the blocking step, four-times the volume (v/v) of HENS (225 mM HEPES-NaOH pH 7.2, 0.9 mM EDTA, 0.1 mM Neocuproine, 2.5% (w/v) SDS) was freshly prepared, and 30 mM NEM was added to the protein extracts; the samples were incubated at 37°C for 30 min. Excess NEM was removed by precipitation with ice-cold acetone, and the protein pellet was re-suspended in 0.5 ml HENS buffer (without NEM) per milligram of protein in the starting sample. Biotinylation was achieved by adding biotin-HPDP and SIN (1 mM and 3 mM final concentrations, respectively) with further incubation at room temperature for 1 hour in the dark. The controls for false-positive signals (FP) were treated with SIN in the presence of NEM for 25 min at 37°C before the biotinylation step. After biotinylation, the proteins were precipitated with acetone and subjected to Western blot analyses and/or affinity purification of biotinylated proteins by NeutrAvidin agarose as described elsewhere [11]. For Western blot analyses, protein pellets were re-suspended in sample buffer and were separated by non-reducing SDS-PAGE on 12% polyacrylamide gels followed by immunoblotting [61]. After immunoblotting, membranes were stained with PonceauS to control protein loadings (Figure S3). After blocking membranes with 1% (w/v) nonfat milk powder and 1% (w/v) bovine serum albumin, the blots were incubated with the anti-biotin mouse monoclonal antibody conjugated with alkaline phosphatase (Sigma-Aldrich, St. Louis, USA; final dilution 1∶10 000) overnight at 4°C. The protein bands were visualized using 5-bromo-4-chloro-3-indolyl phosphate and nitro blue tetrazolium. For affinity purification of biotinylated proteins, the precipitated proteins were re-suspended in HENS buffer (100 µl per mg of protein in the starting sample) and 2 volumes of neutralization buffer (20 mM HEPES, pH 7.7, 100 mM NaCl, 1 mM EDTA, and 0.5% (v/v) Triton X-100). Biotinylated proteins were incubated for 1 hour at room temperature with the NeutrAvidin-agarose (30 µl per mg of protein). The agarose-matrix was washed extensively with 20 volumes of washing buffer (600 mM NaCl in neutralization buffer) and bound proteins were eluted with 100 mM β-mercaptoethanol in elution buffer (20 mM HEPES, pH 7.7, 100 mM NaCl, 1 mM EDTA) and precipitated with ice-cold acetone.

### In-solution digest of S-nitrosylated proteins after NeutrAvidin affinity purification

The pellets from the acetone-precipitation (section before) were dissolved in 30 µl of 50 mM ammonium bicarbonate (AmBic). For protein reduction, 2 µl of 100 mM DTT was added and incubated for 15 min at 60°C. After cooling to room temperature, the free cysteine residues were alkylated by adding 2 µl of freshly prepared 300 mM iodoacetamide (IAA) solution for 30 min in the dark. A tryptic digest was performed overnight at 37°C using 0.5 µg of trypsin (Promega, Mannheim, Germany) per sample. To stop the digest, the sample was acidified using trifluoroacetic acid (TFA) and then stored at −20°C.

### Preparation of whole-cell extracts (WCE) for overall proteomic analyses

For normalization of the protein abundance of S-nitrosylated proteins, we determined the total protein abundances by preparing WCE of C and O samples. Fifty mg of frozen leaf powder was mixed with 1 ml HENT buffer (for buffer composition see above) containing a protease inhibitor cocktail tablet and incubated on ice for 10 min. After centrifugation for 10 min (14 000 *g*), the protein extract was passed through a Sephadex G-25 column (GE Healthcare, München, Germany) using HEN (without Triton X-100) as a buffer. The protein concentration was determined by the Bradford assay with bovine serum albumin (BSA) as a standard.

### Filter aided proteome preparation (FASP) digest of proteins from WCEs

Of each of three WCEs of C and O samples, an aliquot containing 10 µg of protein was digested using a modified FASP procedure [62]. In brief, the proteins were reduced and alkylated using DTT and IAA and then centrifuged through a 30 kDa cut-off filter device (PALL, Port Washington, USA), washed thrice with UA buffer (8 M urea in 0.1 M Tris/HCl pH 8.5) and twice with 50 mM AmBic. The proteins were digested for 2 hours at room temperature using 1 µg Lys-C (Wako Chemicals, Neuss, Germany) and for 16 hours at 37°C using 2 µg trypsin (Promega, Mannheim, Germany). The peptides were collected by centrifugation (10 min at 14 000 *g*), and the samples were acidified with 0.5% TFA and stored at −20°C.

### Mass spectrometry

Digested samples (after affinity purification or from WCE) were thawed and centrifuged (14 000 *g*) for 5 min at 4°C. The LC-MS/MS analysis was performed as previously described [63]. Every sample was automatically injected and loaded onto the trap column at a flow rate of 30 µl min^−1^ in 5% buffer B (98% acetonitrile (ACN)/0.1% formic acid (FA) in HPLC-grade water) and 95% buffer A (2% ACN/0.1% FA in HPLC-grade water). After 5 min, the peptides were eluted from the trap column and separated on the analytical column by a 170 min gradient from 5 to 31% of buffer B at 300 nl min^−1^ flow rate followed by a short gradient from 31 to 95% buffer B for 5 min. Between each sample, the gradient was set back to 5% buffer B and left to equilibrate for 20 min. From the MS pre-scan, the 10 most abundant peptide ions were fragmented in the linear ion trap if they showed an intensity of at least 200 counts and if they were at least +2 charged. During fragmentation a high-resolution (6×10^4^ full-width half maximum) MS spectrum was acquired in the Orbitrap (Thermo Fischer Scientific, Bremen, Germany) with a mass range from 200 to 1 500 Da.

### Label-free analysis using Progenesis LC-MS

The acquired spectra were loaded to the Progenesis LC-MS software (v2.5, Nonlinear Dynamics Ltd, Newcastle upon Tyne, UK) for label-free quantification and analyzed as previously described [63], [64]. Features of only one charge or more than eight charges were excluded. The raw abundances of the remaining features were normalized to allow for the correction of factors resulting from experimental variation. Rank 1–3 MS/MS spectra were exported as a MASCOT generic file and used for peptide identification with MASCOT (v2.2 and 2.3.02, Matrix Science, London, UK) in the *Populus trichocarpa* protein database (v4; 17 236 452 residues; 45 036 sequences). The search parameters were 10 ppm peptide mass and 0.6 Da MS/MS tolerance, one missed cleavage allowed.

For the identification and quantification of S-nitroso-proteins, N-ethylmaleinimidation and carbamidomethylation were set as variable modifications, as well as methionine oxidation. A MASCOT-integrated decoy database search calculated a false discovery rate (FDR) of 0.17% using a MASCOT ion score cut-off of 30 and a significance threshold of *P*<0.01.

For the identification and quantification of total proteins in the WCEs of leaves, carbamidomethylation was set as a fixed modification, and methionine oxidation and deamination of asparagine/glutamine as variable modification. A MASCOT-integrated decoy database search calculated a FDR of <1%. The MASCOT Percolator algorithm was used to distinguish between correct and incorrect spectrum identification [65], with a maximum *q* value of 0.01. The peptides with a minimum percolator score of 15 were used further.

For each dataset, the peptide assignments were re-imported into the Progenesis LC-MS software. After summing up the abundances of all of the peptides that were allocated to each protein, the identification and quantification results were exported and are given in Table S1.

### Statistics

The differences in the S-nitroso-proteome between control and ozone-treated samples were analyzed as previously described [66] using Principal Component Analysis (PCA) and Orthogonal Partial Least Square regression (OPLS) statistical methods from the software packages ‘SIMCA-P’ (v13.0.0.0, Umetrics, Umeå, Sweden). The results were validated by ‘full cross validation’ [67] using a 95% confidence level.

Before PCA and OPLS, S-nitroso-protein intensities were normalized to ensure that quantitative differences in S-nitrosylation between the C and O samples were not due to different amounts of the respective proteins. The normalization process and the calculated log2-fold changes (ozone/control) are given in Table S1C. In brief, the abundance of each S-nitrosylated protein (yellow, C1, C2, etc.) was normalized to the corresponding (averaged) protein abundance in the whole-cell extracts (WCE) of the C and O leaves (i.e. green, Avg_C_total, Avg_O_total). For the very lowly abundant proteins, which were not detected in the WCEs (25 proteins out of 172 proteins, highlighted in red), the total protein abundance was calculated based on the fraction of S-nitroso-protein content over the total protein content in the WCE detected protein, i.e multiplying by 100 and dividing by the median (i.e. 2.2) of percentages between all S-nitroso-proteins and the respective measured total proteins. PCA was performed on normalized, summed S-nitroso-protein intensities (centered and scaled with 1 SD^−1^) used as X-variables. X-data were pre-processed by logarithmic (base 10) transformation. Three independent biological replicates were used for each C and O treatment and their respective FP samples were included as controls of the biotin switch assay. Therefore, the size of the analyzed matrix was 172-by-8.

OPLS was employed to understand how the pattern of S-nitrosylated proteins changed upon ozone treatment and to discover which S-nitroso-proteins are affected by ozone exposure. OPLS was performed as PCA and by giving as Y-variable the value of 0 to C samples and value of 1 to O samples. S-nitroso-proteins showing Variable of Importance for the Projection (VIP) greater than 1 and uncertainty bars of jack-knifing method [68] smaller than the respective VIP value were defined as discriminant proteins that can separate O from C samples. Additionally, discriminant proteins were tested for significance difference (*P*<0.05) between C and O samples independently from multivariate data analysis using Student’s *t*-test and applying a FDR of 5% according to the Benjamini Hochberg modified correction (MATLAB R2011b; MathWorks, Natick, USA) [69], [70]. PCA and OPLS were also performed on non-normalized abundances, giving similar results.

### Determination of nitrite and nitrosothiol content

The quantification of nitrite and nitrosothiols (SNO) in poplar leaf tissue was performed using Sievers’ Nitric Oxide Analyzer (NOA 280i, GE Water & Process Technologies, Ratingen, Germany). The instrument is based on ozone-dependent chemiluminescence: in the reaction vessel, nitrite and SNO are reduced to NO, which reacts with ozone to yield light photons. A total of 200 µg frozen plant powder from leaf tissue (C and O) was mixed with 600 µl extraction buffer (1×PBS pH 7.7, 10 mM NEM, 2.5 mM EDTA) and centrifuged twice (14 000 *g* for 20 min). For nitrite and SNO determination, 100 µl and 200 µl of the plant extracts, respectively, were injected into the reaction vessel of the NOA 280i containing the reducing agent iodine, iodide and acetic acid at room temperature. To measure the SNO content, the plant extracts were pre-treated in a mixing ratio of 9∶1 with 5% sulfanilamide (w/v, in 1 M HCL) to chemically remove nitrite. The peak area integration and quantification of nitrite and SNO contents were performed with Sievers NO Analysis Software (v3.2) using nitrite standards.

### Determination of PAL activity

The assay for the measurement of the PAL activity was modified from Heide et al. [71]. The WCEs from C and O leaves were used as the starting material. To measure the effect of S-nitrosylation and de-nitrosylation, the WCEs were pre-treated with either GSNO (500 µM) or SIN (3 mM), respectively, for 1 hour at room temperature in the dark. GSNO is a widely used NO donor leading to enhanced S-nitrosylation levels of proteins [11], [13], [72]. The inhibitory effect of GSNO treatment on the enzyme activity has been shown for several proteins [10]. SIN is used as de-nitrosating agent in the biotin switch assay [59]. The PAL activity assay began by mixing 150 µl of pre-treated WCE, 150 µl of 0.2 M sodium borate buffer (pH 8.9) and 100 µl L-phenylalanine (in sodium borate buffer). The reaction was performed at 37°C for 2 hours and was stopped by adding 100 µl of 6 N HCl. The controls were run without adding L-phenylalanine or WCE. The reaction product *trans*-cinnamic acid was extracted with 500 µl ethyl acetate, stirred for 15 min and then centrifuged for 2 min at 14 000 *g*. The ethyl acetate layer was transferred to a new tube and evaporated. The residue was dissolved in 150 µl methanol and 100 µl double distilled water and analyzed by HPLC using the Beckman Gold 7.11 HPLC system (Beckman Coulter, Krefeld, Germany) with a Bischoff ProntoSIL Spherisorb ODS2 Type NC separation column (5 µm–250 mm×4.6 mm) (Bischoff Chromatography, Leonberg, Germany) at a flow rate of 1 ml min^−1^. The sample injection volume was 10 µl, solvent A was water and 5% ammonium formate in formic acid (mixed at a ratio of 98∶2, respectively) and solvent B methanol, double distilled water and ammonium formate in FA (mixed at a ratio of 88.2∶9.8∶2, respectively). The separation program was isocratic with 60% solvent B for 2 min, linear gradient to 100% solvent B for 5 min, 100% solvent B for 4 min, linear gradient to 60% solvent B for 1 min and isocratic with 60% solvent B for 3 min. The absorbance at 273 nm was used for detection. The specific enzyme activity was calculated using the total protein concentration of WCEs.

## Results

### Detection of *in vivo* S-nitrosylated proteins in green tissues of poplar

We aimed to survey proteins that are constitutively S-nitrosylated in poplar callus and leaf tissues. Therefore, protein extracts from grey poplar callus and leaves from control (C) and ozone-treated (O) plants were subjected to the biotin switch assay and Western blot analysis. Several bands were detected in callus and leaf extracts compared to controls (FP), indicating the presence of S-nitrosylated proteins *in vivo* (Figure S3). The most prominent band in the Western Blot of the leaf tissue was the ribulose-1,5-bisphosphate carboxylase/oxygenase (RuBisCO) large subunit (∼50 kDa), a well-known target of S-nitrosylation in plants [11], [24]–[26]. In the callus tissue, which was only slightly green (Figure S1), the amount of RuBisCO is decreased compared to that in the leaf samples, as indicated by the weaker Ponceau S-stained protein band on the gel (Figure S3). No bands were visualized on the Western blot of False Positive (FP) samples, the controls of biotin switch assay.

**Figure 1:**
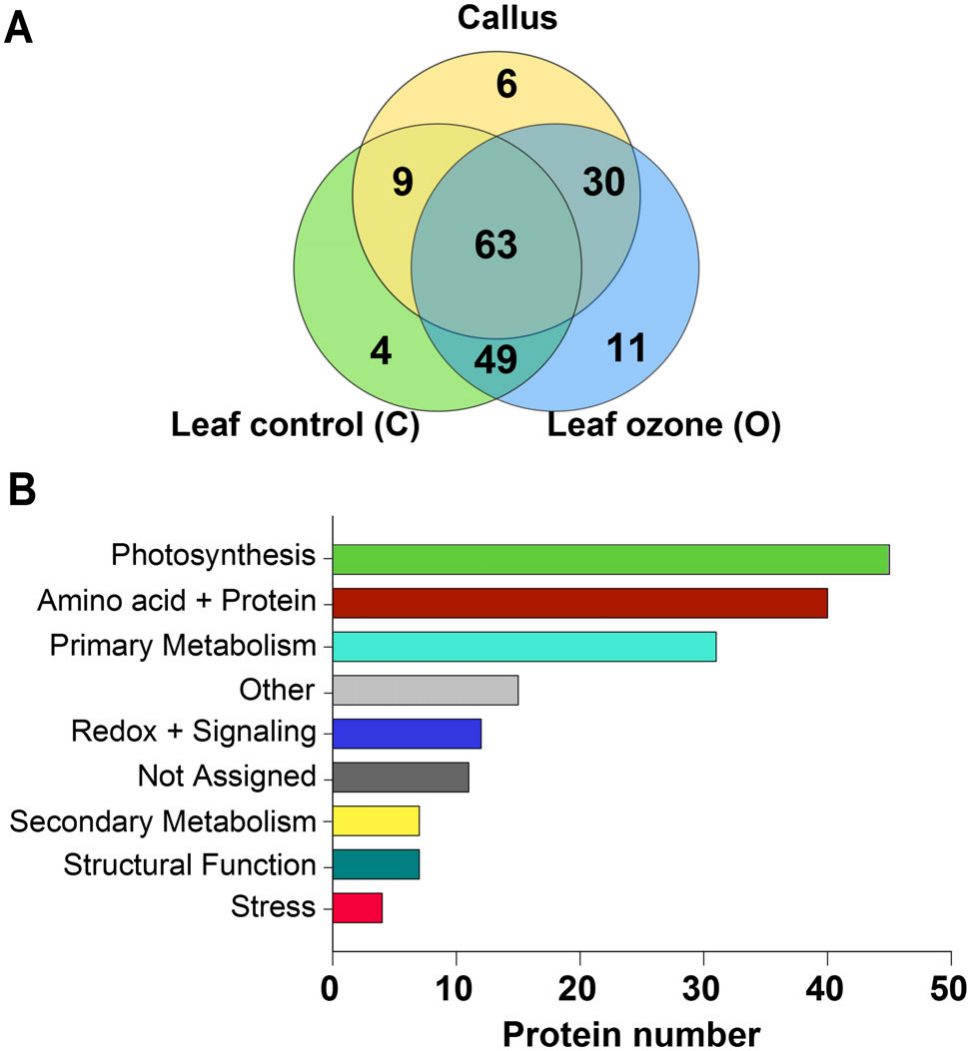
Identification of endogenously S-nitrosylated proteins in grey poplar. (A) VENN diagram visualizing the number of identified S-nitrosylated proteins by LC-MS/MS in callus and leaf tissue (C and O) of poplar. (B) Functional categorization of the 172 identified S-nitrosylated proteins in poplar callus and leaf samples according to MapManBINs (http://ppdb.tc.cornell.edu/dbsearch/searchacc.aspx).

LC-MS/MS analysis of NeutrAvidin affinity chromatography-purified proteins allowed the identification (MASCOT ion scores >30 and protein identifications based on at least two unique peptides) of a total of 172 S-nitrosylated proteins (Table S1A). About one-third of the proteins (63 proteins) were common in all samples (callus, leaf C and leaf O), whereas 11 of the remaining 109 proteins were exclusively detected in ozone-treated leaves, 6 in the callus and 4 in control leaves (Figure 1A). To functionally categorize the proteins, a MapManBIN search (http://ppdb.tc.cornell.edu/dbsearch/searchacc.aspx) was performed using the accession numbers of the respective Arabidopsis orthologs (Figure 1B, Table S2). The 172 S-nitrosylated proteins were clustered in 9 main functional categories according to MapManBIN. ‘Photosynthesis’ represented the main group (26% of the total number of proteins), comprising the photosynthetic light reactions, Calvin cycle, photorespiration and enzymes of the tetrapyrrole synthesis pathway. ‘Amino acid + Protein’ represented the next frequent group (23%) containing enzymes of the amino acid metabolism and the protein synthesis, degradation and folding process. 18% of the proteins were associated to ‘Primary metabolism’. These are amongst others aconitase (EC 4.2.1.3) and malate dehydrogenase (EC 1.1.1.37) of the citric acid cycle; ribulose-5-phosphate-3-epimerase (EC 5.1.3.1), transketolase (EC 2.2.1.1), and transaldolase (EC 2.2.1.2) of the pentose phosphate pathway and eight glycolytic enzymes, with phosphofructokinase (EC 2.7.1.11) and pyruvate kinase (EC 2.7.1.40) regulating two committing steps in glycolysis. Ribulose-5-phosphate-3-epimerase of the pentose phosphate pathway was identified for the first time as target of S-nitrosylation in plants. Proteins assigned to ‘Redox + Signaling’, ‘Secondary Metabolism’ and ‘Structural function’ represented 7%, 4% and 4%, respectively. A total of 6% of the proteins could not be functionally annotated or are unknown proteins. Categories containing less than four proteins were summarized in ‘Other’ (9%). Our MS analysis revealed 43 not previously described candidates for S-nitrosylation in plants, including six enzymes of the polyphenol biosynthetic pathways: phenylalanine ammonia-lyase (EC 4.3.1.24; PAL2 and PAL3), chalcone synthase (EC 2.3.1.74; CHS), naringenin 3-dioxygenase (EC 1.14.11.9; F3H), caffeic acid 3-O-methyltransferase (EC 2.1.1.68; COMT) and polyphenol oxidase (EC 1.14.18.1; PPO) (see Table S1A, highlighted in bold).

### Acute ozone stress evokes rapid changes in the S-nitroso-proteome of poplar

**Figure 2:**
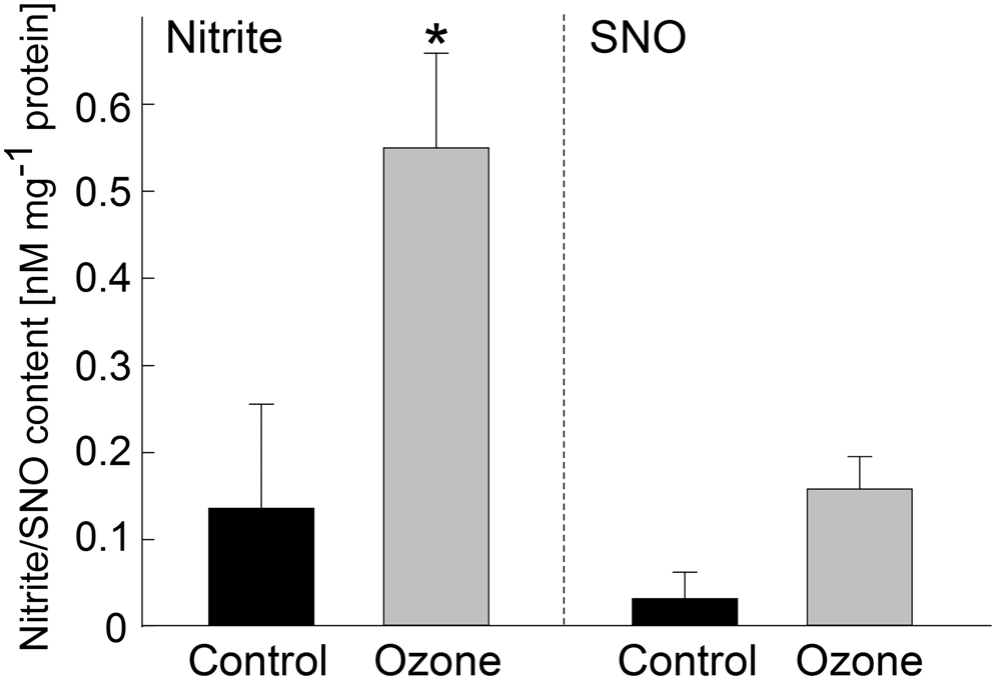
Nitrite and nitrosothiol (SNO) contents in grey poplar leaves upon acute ozone exposure (grey bars) compared to controls (black bars). Poplar plants were fumigated with ozone and harvested immediately after ozone treatment. 200 µg homogenized plant material was mixed with 600 µl PBS buffer containing NEM and EDTA. For nitrite and SNO determination, 100 µl and 200 µl of the plant extracts were, respectively, injected into the reaction vessel of Sievers NO analyzer containing the reducing agent tri-iodide. To measure SNO, the plant extracts were pre-treated with sulfanilamide to chemically remove nitrite. Values represent means ± SE of 3 biological replicates measured in three independent analyses. *P<0.05 (Student’s *t-*test).

Acute ozone fumigation has been shown to induce transient NO production in Arabidopsis and tobacco plants [40], [42], [43], [73]. Using a chemiluminescence-based assay, we measured the nitrosothiol (SNO) and nitrite contents in control and ozone-treated poplar leaves. After 1 hour of ozone exposure the nitrite content in O leaves was 4-fold higher and the SNO content was 3.5-fold higher, although only the nitrite content was found to be statistically different (*P*<0.05, Students *t*-test) (Figure 2).

**Figure 3:**
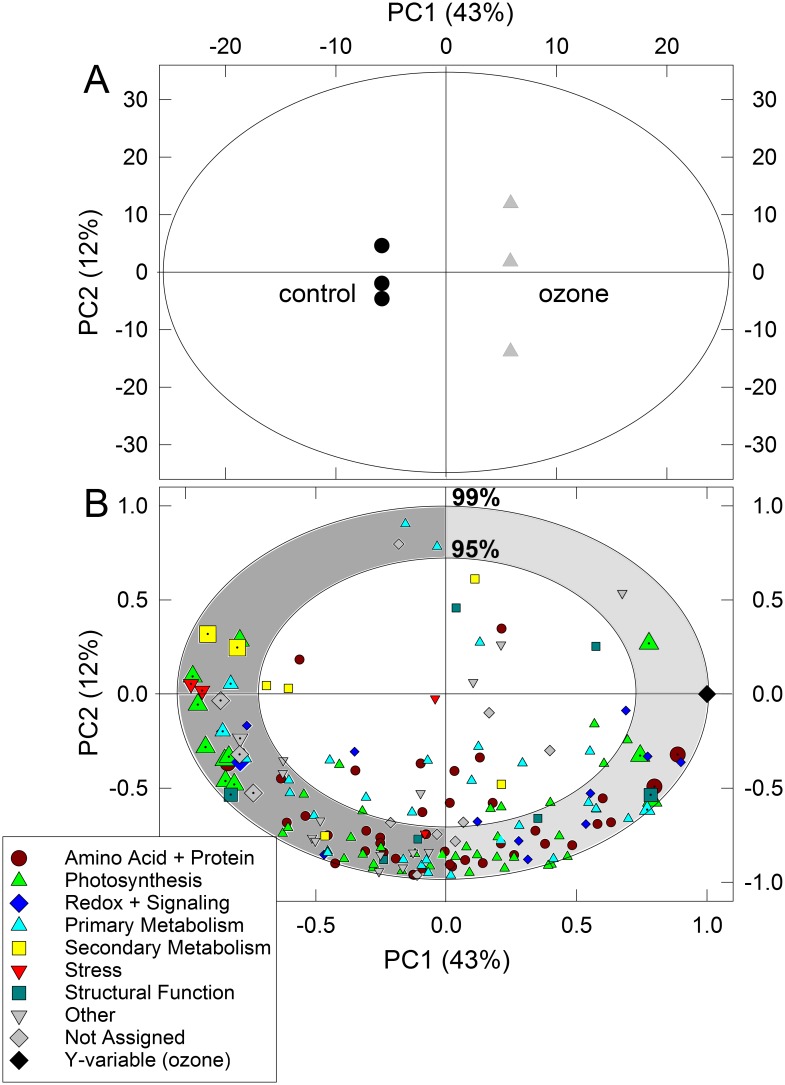
Two-dimensional (A) score and (B) scaled and centered loading plots of orthogonal partial least square regression (OPLS) of S-nitrosylated proteins (normalized by whole-cell extract) in poplar leaf samples determined using biotin switch assay and LC-MS/MS. The explained variance (in percentage) and the number of principal components (PC) are reported in the x- and y-axes in both (A) and (B) plots. The ellipse in (A) indicates the tolerance based on Hotelling’s *T^2^* with the significance level of 0.05. The outer and inner ellipses in (B) indicate 100% and 75% explained variance, respectively. (A) control = black circles, ozone-treated = grey triangles; (B) each functional group of proteins (according to Figure 1C) is indicated with different symbols, zoomed symbols with a dot represent the significantly different proteins between C and O plants tested independently with Student’s *t-*test (*P*<0.05 applying a FDR of 5%). Symbol legend: dark red circles = Amino acid metabolism and Protein synthesis, folding and degradation; blue diamonds = Redox and Signaling; cyan triangles-up = Primary metabolism; yellow square = Secondary metabolism; red triangles-down = Stress; dark green squares = Structural function; grey triangles-down = other; grey squares = not assigned or not identified. The black diamond indicates the Y-variable, i.e. ozone-treatment.

To gain insight into the change of the S-nitrosylation signaling during ozone stress, we performed a quantitative proteomic analysis (label-free LC-MS/MS). Principal component analysis (PCA) revealed a clear ozone-induced change in the S-nitrosylated protein profile of C and O leaves, as indicated by the separation of C from O samples along the second principal component (PC2) (Figure S4). The first component (PC1) accounts for most of the explained variance (57%) and shows large differences between samples containing S-nitrosylated proteins and their false-positive controls (FP-samples in Figure S4), indicating a reliable detection of S-nitrosylated proteins using the biotin switch assay and LC-MS/MS. OPLS was further employed for studying the S-nitroso-protein patterns of C and O samples in greater detail, and for evaluating to what extent the S-nitrosylated proteins became up- and down-regulated upon ozone exposure. The O samples were separated from the C samples by 43% explained variance of the PC1 (Figure 3A). The proteins that statistically discriminate between C and O samples are summarized in Table 1 together with the corresponding log2-fold changes and the additional statistical analysis (*t*-test). The analysis revealed that the content of 27 and 13 S-nitrosylated proteins were decreased and increased upon ozone fumigation, respectively. Among those 40 negatively and positively correlated proteins to ozone treatment, 32 proteins significantly changed at *P*<0.05 (*t*-test; FDR 5%).

**Table 1:** Variable Importance for the Projection (VIP) for each discriminant protein that separates ozone fumigated poplar leaves (O) from control leaves (C) in the OPLS model.

Functionalcategory	Accession	VIP score	SE	Description	Function (MapManBIN)	Log2 (O/C)
**Decrease in S-nitrosylation after ozone**						
**Amino acid+** **Protein**	POPTR_0017s10920.1	1.721	0.797	Cyclophylin-type protein	Protein/folding	−1.3*
	POPTR_0021s00380.1	1.256	1.202	T-protein of glycine decarboxylasecomplex	Amino acid metabolism/degradation	−0.6
**Photosynthesis**	POPTR_0001s13800.1	1.997	0.513	Chlorophyll a/b-binding protein 2precursor	Photosynthesis/lightreaction	−2.5**
	POPTR_0015s07340.1	1.955	0.591	Chlorophyll a/b-binding protein 4	Photosynthesis/lightreaction	−2.3**
	POPTR_0005s26080.1	1.896	0.510	Chlorophyll a/b-binding protein 2	Photosynthesis/lightreaction	−1.6**
	POPTR_0001s11600.1	1.745	0.702	Photosystem I reaction centersubunit III	Photosynthesis/lightreaction	−1.4*
	POPTR_0019s09140.1	1.738	0.827	Chlorophyll a/b-binding protein CP26	Photosynthesis/lightreaction	−1.5*
	POPTR_0011s02770.1	1.710	0.998	Chlorophyll a/b-binding protein 2	Photosynthesis/lightreaction	−3.7*
	POPTR_0010s22790.1	1.669	0.952	Chlorophyll a/b-binding protein,putative	Photosynthesis/lightreaction	−3.5*
	POPTR_0008s15100.1	1.627	1.103	Photosystem I 20 kD protein	Photosynthesis/lightreaction	−0.5*
	POPTR_0008s06720.1	1.288	1.213	Chlorophyll a/b-binding protein	Photosynthesis/lightreaction	−0.8
**Primary** **Metabolism**	POPTR_0018s07380.1	1.760	0.874	Sucrose synthase	Major CHO metabolism/degradation	−3.0*
	POPTR_0018s09380.1	1.697	1.007	Malate dehydrogenase	TCA	−1.3*
	POPTR_0012s09570.1	1.593	1.091	Glyceraldehyde-3-phosphatedehydrogenase	Glycolysis	−0.6*
	POPTR_0008s05640.1	1.233	1.229	Triosephosphate isomerase, cytosolic	Glycolysis	−0.7
**Secondary** **Metabolism**	POPTR_0012s00670.1	1.877	0.817	Caffeic acid 3-O-methyltransferase	Secondary metabolism/phenylpropanoids	−3.4**
	POPTR_0008s03810.1	1.644	1.107	Phenylalanine ammonia-lyase 2	Secondary metabolism/phenylpropanoids	−3.6*
	POPTR_0001s39630.1	1.415	1.222	Polyphenol oxidase	Secondary metabolism	−2.0
**Stress**	POPTR_0019s12370.1	2.010	0.496	EP3 chitinase	Stress/biotic	−4.7**
	POPTR_0018s10490.1	1.922	0.775	Thaumatin, pathogenesis-relatedprotein	Stress/abiotic	−3.8**
**Structural** **Function**	POPTR_0016s02620.1	1.696	0.805	Alpha-N-arabinofuranosidase	Cell wall	−1.0*
**Redox +** **Signaling**	POPTR_0006s11570.1	1.774	1.004	Monodehydroascorbate reductase	Redox/ascorbate and glutathione	−0.7*
	POPTR_0004s10120.1	1.570	1.172	Multifunctional chaperone (14-3-3family)	Signaling	−0.3*
**Other**	POPTR_0018s09580.1	1.622	1.146	GDSL-like lipase/acylhydrolase	Misc/GDSL-motif lipase	−1.1*
**Not Assigned**	POPTR_0005s17350.1	1.777	0.978	Ascorbate peroxidase, putative	Not assigned	−0.9*
	POPTR_0005s22860.1	1.627	1.106	Aldo/keto reductase family protein	Not assigned	−0.8*
	POPTR_0002s05640.1	1.518	1.148	Aldo/keto reductase family protein	Not assigned	−1.0*
**Increase in S-nitrosylation after ozone**						
**Amino acid+** **Proteins**	POPTR_0002s12130.1	1.824	0.889	Aconitase	Protein/degradation	1.0**
	POPTR_0003s20870.1	1.640	1.017	Chaperonin precursor	Protein/folding	1.6*
**Photosynthesis**	POPTR_0007s07680.1	1.623	1.062	Porphobilinogen deaminase	Tetrapyrrole synthesis	2.6*
	POPTR_0003s08760.1	1.595	1.050	Glycine cleavage system protein H precursor	Photosynthesis/photorespiration	1.4*
	POPTR_0015s07330.1	1.528	1.066	Ribulose-phosphate 3-epimerase	Photosynthesis/calvincycle	1.0*
	POPTR_0014s11580.1	1.427	1.300	CP12 domain-containing protein	Photosynthesis/calvincycle	1.9
**Primary** **Metabolism**	POPTR_0006s03660.1	1.583	1.151	Glutamate synthase, ferredoxin-dependent	N-metabolism	1.3*
	POPTR_0016s03630.1	1.434	1.275	Glutamate synthase, ferredoxin-dependent	N-metabolism	1.9
**Redox+** **Signaling**	POPTR_0001s22980.1	1.848	0.869	Calmodulin-like protein 6a	Signaling/calcium	2.9**
	POPTR_0013s10250.1	1.586	1.077	Peroxiredoxin 5, putative	Redox	0.8*
	POPTR_0002s08260.1	1.416	1.110	Protein disulfide-isomerase,precursor	Redox	0.7
**Structural** **Function**	POPTR_0001s29670.1	1.611	1.055	Tubulin alpha chain	Cell/organisation	1.1*
**Other**	POPTR_0001s23310.1	1.386	1.226	Glycosyl hydrolase familyprotein	Misc/gluco-, galacto- andmannosidases	1.0

Proteins showing VIP >1 and uncertainty bars of jack-knifing method (SE) < than the respective VIP value were defined as discriminant proteins. Additionally, discriminant proteins were tested for significance difference (*P*<0.05) between C and O plants independently from multivariate data analysis using *t-*test (* = *P*<0.05; ** = *P*<0.01) applying a FDR of 5%. Log fold changes (ozone/control) were calculated from normalized protein abundances (Table S1C). Annotation and functional categorization was obtained from Phytozome and PPDB (http://www.phytozome.net/; http://ppdb.tc.cornell.edu/dbsearch/searchacc.aspx).

Specifically, O samples were negatively correlated to proteins of the secondary metabolism (see yellow squares in Figure 3B). Three enzymes of the secondary metabolism became de-nitrosylated in O samples as seen by the negative fold changes. The S-nitrosylation level of COMT (log2(O/C) = −3.4) and PAL2 (−3.6) was significantly lower in the O samples (*P*<0.05) whereas log fold changes <−1 but no statistical significance was found for PPO (log2 = −2.0; *t-*test, *P* = 0.064). Another group of proteins affected by ozone was related to photosynthesis (Figure 3B, green triangles), mostly comprising proteins of the light-harvesting complex (chlorophyll a/b-binding proteins). Moreover, a significant decrease in S-nitrosylation was observed in the following: cyclophylin-type protein (−1.3), sucrose synthase (−3.0; EC 2.4.1.13), malate dehydrogenase (−1.3; EC 1.1.1.37), glyceraldehyde-3-phosphate dehydrogenase (−0.6; EC 1.2.1.59), stress-related EP3 chitinase (−4.7; EC 3.2.1.14), thaumatin (pathogenesis-related protein; −3.8), alpha-N-arabinofuranosidase (−1.0; EC 3.2.1.55), monodehydroascorbate reductase (−0.7; EC 1.6.5.4), multifunctional chaperone (14-3-3 family; −0.3), GDSL-like lipase/acylhydrolase (−1.1), ascorbate peroxidase (−0.9; EC 1.11.1.11) and two aldo/keto reductase family proteins (−0.8; −1.0).

**Figure 4:**
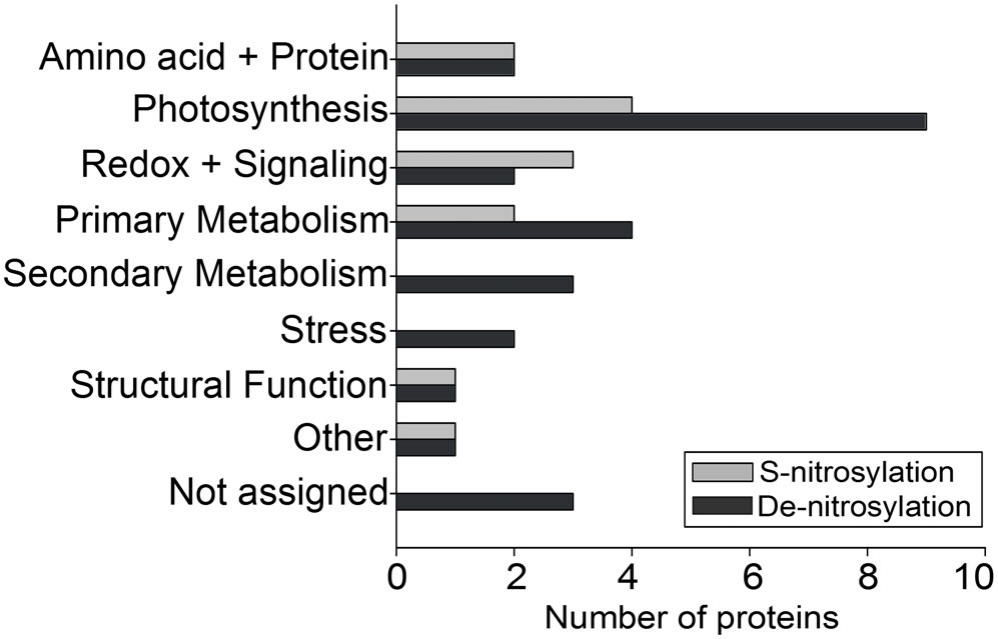
Number of proteins with increased (grey bars) or decreased (dark grey bars) level of S-nitrosylation in O samples of grey poplar according to Table 1. The proteins were grouped based on their function.

The proteins which positively correlated to O samples in the OPLS are functionally categorized into ‘Amino acid+Proteins’, ‘Photosynthesis’, ‘Primary Metabolism’, ‘Redox+Signaling’, ‘Structural Function’ and ‘Other’ (Table 1, Figure 4). Calmodulin-like protein 6a and peroxiredoxin 5 show a significant increase in S-nitrosylation after ozone fumigation compared to the C samples (log2 = +2.8 and +0.9, respectively) as well as aconitase (+1.0), chaperonin precursor (+1.6), porphobilinogen deaminase (+2.6; EC 2.5.1.61), glycine cleavage system protein H precursor (+1.4), ribulose-phosphate 3-epimerase (+1.0; EC 5.1.3.1), glutamate synthase (+1.3; EC 1.4.7.1) and tubulin alpha chain (+1.1). Taken together, one hour of ozone fumigation dramatically changed the S-nitrosylation status of leaf proteins, inducing rapid de-nitrosylation and S-nitrosylation events in 32 proteins.

### Acute ozone stress and de-nitrosylation increase PAL enzyme activities

**Figure 5:**
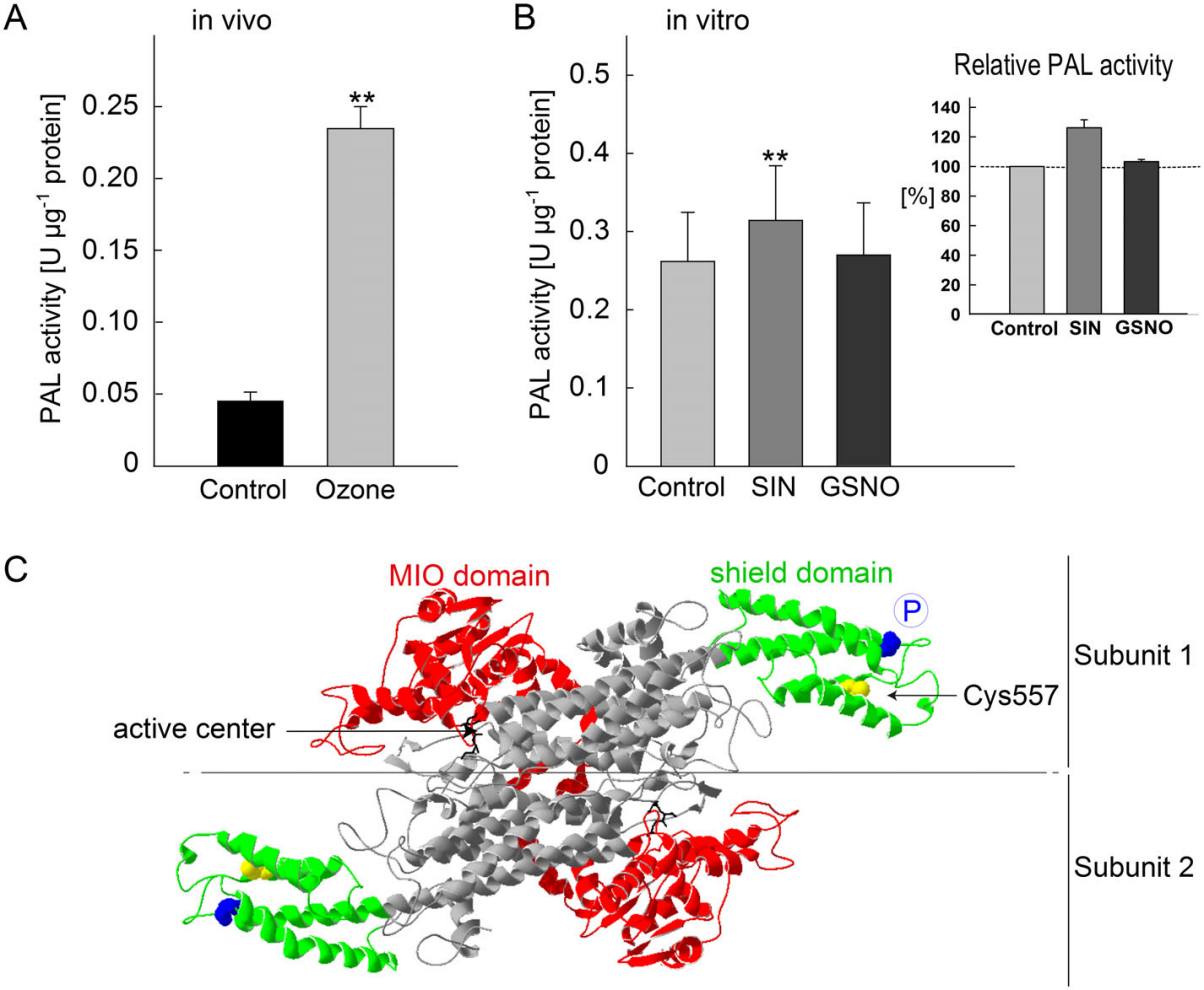
Effect of SIN, GSNO and ozone on the activity of PAL in grey poplar leaves. (A) The PAL activity was analyzed at the end of ozone fumigation or in control samples at the same time point. The values represent the means ± SE of three experiments. **P<0.01 (Student’s t-test for unpaired samples). (B) Effect of SIN (removal of NO groups) and GSNO (NO-donor) on *in vitro* PAL activity. 12-week-old plants grown under greenhouse conditions were used as leaf material. Before measuring the enzyme activity, the leaf extracts were pre-incubated for 1 hour with 3 mM SIN or 500 µM GSNO. Each value represents the mean of nine replicates ± SE and significant differences are given with ** for *P*<0.01 (Student’s *t*-test for paired samples). The relative PAL activity was calculated by comparing each replicate in the SIN and GSNO group to the corresponding value in the control group. The PAL activity in the control was taken as 100%. (C) Structural model of PAL2 from *Populus trichocarpa*. The three-dimensional structure was modeled according the crystal structure of *Petroselinum crispum* (PDB code: 1w27B) using SWISS-MODEL [125], [126]. For clarity, a dimer of the actual tetrameric PAL2 is shown. The flexible shield domain is highlighted in green and contains the cysteine predicted to be S-nitrosylated in PAL2 (Cys557; yellow) and the postulated phosphorylation site at Thr549 (blue). The MIO domain (red) contains the catalytic active center (black).

To demonstrate the effect of S-nitrosylation on an enzyme activity, we chose to analyze PAL, which is the first regulatory enzyme in the phenylpropanoid pathway of plants. It has been shown that short pulses of ozone (duration <10 hours) increase both PAL activity and PAL gene expression [44], [74], [75]. Here, we measured a 5-fold increase in PAL activity compared to that in untreated controls after 1 hour of ozone treatment (Figure 5A).

The *Populus trichocarpa* protein database comprises five isoenzymes of PAL (PAL1 to PAL5; Figure S5). Applying the biotin-switch assay, PAL2 and PAL3 were identified as candidates for S-nitrosylation (Table S1A) and PAL2 was revealed as significantly de-nitrosylated after ozone exposure (Table 1). To determine if de-nitrosylation affected the overall PAL enzyme activity, we measured the PAL activities of leaf extracts *in vitro* after incubation with sinapic acid (SIN) or GSNO (Figure 5B). SIN caused a significant (*P*<0.01, paired *t*-test) increase in PAL activities, whereas GSNO did not, indicating that de-nitrosylation positively regulates PAL activity. The relative increase of PAL activity was approx. 25% (Figure 5B; insert).

The alignment of the amino acid sequences of the five PAL isoenzymes of *Populus trichocarpa* with PAL sequences from Arabidopsis, tobacco, parsley and pea revealed highly conserved cysteine residues (Figure S5). Of these cysteine residues, two were predicted by the GPS-SNO software to be S-nitrosylation sites in *Populus trichocarpa* PAL2 (Cys557 and Cys691) [76]. As shown in Figure 5C, Cys557 of PAL2 is located within the shield domain (highlighted in green). This domain narrows the substrate access to a small tunnel and is highly mobile [77]. Also in the shield-domain, a phosphorylation site was detected in the PAL of French bean (Thr545) [78]. This amino acid is also highly conserved in the amino acid sequence of poplar PAL (Figure S5). Phosphorylation at Thr545 (at the transition between the core domain and shield domain) might induce conformational changes in the flexible loop of the shield domain of each subunit (functional PAL is a tetramer of identical subunits), thereby changing the accessibility of the substrate to the active site of neighboring units.

## Discussion

### Homeostatic level of S-nitrosylated proteins in poplar green tissues

This study represents the investigation of protein S-nitrosylation patterns in the woody model plant grey poplar. By performing a biotin switch assay optimized for poplar in conjunction with the quantitative LC-MS/MS analysis (label-free), we identified 172 proteins that are S-nitrosylated *in vivo* in callus tissue and fully developed poplar leaves under unstressed conditions and that appear after a short pulse of oxidative stress. The primary outcome is the observation that key enzymes of the phenylpropanoid and subsequent flavonoid and lignin biosynthetic pathways (PAL2, PAL3, CHS, COMT, F3H and PPO) are possible targets of S-nitrosylation. The polyphenol biosynthesis metabolism generates an enormous collection of secondary metabolites that are involved in plant development and stability (i.e., lignin, suberin, and condensed tannins) [79], plant defense responses against abiotic, and biotic environmental constraints (i.e., stilbenes, flavonoids, and anthocyanins) [80]–[83] and signaling (salicylic acid, and benzoic acids) [84].

Strikingly, many enzymes belonging to the plant primary carbohydrate metabolism (citric acid cycle, glycolysis, pentose phosphate pathway) were identified as targets of S-nitrosylation. S-nitrosylation of the glycolytic enzyme GAPDH results in a reduction of enzyme activity in animals and plants and is a well-known example of how S-nitrosylation negatively affects the enzyme function [12], [85], [86]. It has been proposed that under oxidative stress, the glycolytic pathway is reduced and glucose equivalents are redirected into the pentose phosphate pathway [87]–[89], which is essential for maintaining the cytoplasmic NADPH concentration as a base for the anti-oxidative defense systems [90], [91].

Our proteomic analysis in poplar revealed that photosynthesis is another important cellular process which is regulated by protein S-nitrosylation, supporting observations from previous studies [11], [15], [24], [92], [93]. We observed an increase in the number of S-nitrosylated proteins related to photosynthetic processes when comparing green, un-differentiated calli with fully developed poplar leaves. Various members of the photosynthetic light reaction (e.g., oxygen-evolving complex, proteins of the light-harvesting complex, plastocyanin and thylakoid formation protein (THF)), as well as enzymes of the Calvin cycle (i.e. RuBisCO, RuBisCO activase, phosphoglycerate kinase and aldolase), are S-nitrosylated under steady-state conditions. The THF 1 protein, a new candidate in the context of S-nitrosylation, is a re-modeling factor of Photosystem II (PSII)-Light Harvesting Complex II and is involved in the repair cycle of PSII upon photo damage in Arabidopsis [94]. In addition to the regulation of photochemical aspects, four enzymes of the tetrapyrrole biosynthetic pathway are S-nitrosylated in poplar: glutamate 1-semialdehyde aminotransferase (EC 5.4.3.8), porphobilinogen synthase (syn. aminolevulinate dehydratase; EC 4.2.1.24), porphobilinogen deaminase (syn. hydroxymethylbilane synthase; EC 2.5.1.61) and coproporphyrinogen III oxidase (EC 1.3.3.3). These enzymes catalyze the 3^rd^, 4^th^, 5^th^, and 8^th^ steps of the plastidic porphyrin biosynthesis, respectively [95], providing the backbones for chlorophyll and heme molecules. The identification of porphobilinogen synthase and glutamate 1-semialdehyde aminotransferase as targets of S-nitrosylation is described for the first time. Taken together, the identification of enzymes involved in glycolysis, the pentose phosphate pathway and chlorophyll biosynthesis as targets of S-nitrosylation in plants raises the general question as to how S-nitrosylation is globally involved in the fine tuning of the photosynthetic activity, pigment turnover, Calvin cycle processes and subsequent channeling of the photosynthetic metabolites that are related to the modifications of the glycolytic and pentose phosphate pathway proteins.

### Acute ozone stress alters the homeostasis of S-nitrosylated proteins in poplar

To provide information regarding the biological relevance of S-nitrosylation, the comparative analysis of the S-nitroso-proteome under control and stress conditions is an important tool. At the present, no information is available regarding ozone-induced changes in the S-nitroso-proteome of plants and this is the first study that comprehensively describes it. Here, we applied a short, strong ozone pulse as a model to trigger ROS and NO formation in order to analyze the fast responses in S-nitrosylation/de-nitrosylation.

The cumulative ozone uptake in the present experiment was 110±19 µmol m^−2^, a value of comparable magnitude also applied in earlier studies [57], [58]. We observed no immediate decrease in net CO_2_ assimilation and transpiration rates upon ozone treatment, demonstrating that grey poplar tolerates high acute ozone doses, an observation already mentioned before [58], [96].

Short-term acute ozone fumigation is often used to mimic the HR [35], [40] and therefore the present shifts in the S-nitrosylation pattern might be transferable to early events in leaf pathogenesis. The accumulation of NO and nitrite is a common feature of short-term and chronic ozone fumigation [42], [43], [73]. We observed a rapid nitrite increase and a slight increase in the nitrosothiol (SNO) content in response to the short-term ozone treatment (Figure 2). Increased nitrite content is often observed upon abiotic stresses and is linked to S-nitrosylation events [97]. It has been shown that nitrite induces S-nitrosylation and the subsequent inactivation of the protease caspase-3 [98]. Nitrite, a reservoir for NO, can be reduced back to NO via non-enzyme-dependent reactions [99], [100] or enzymatically by nitrate reductase [101]. Higher levels of SNOs comprise GSNO, a low-molecular weight SNO. GSNO is considered an intracellular NO donor in trans-nitrosylation reactions [102], [103].

Ozone fumigation highly modulated the S-nitroso-proteome. Overall, the S-nitrosylation pattern of 28 proteins was significantly affected. The fact that we observed both S-nitrosylation (one-third of total) and de-nitrosylation (two-thirds of total) events upon ozone stress seems to be contradictory to the accumulation of nitrite and SNO that we measured in response to ozone. However, two other studies [15], [51] also show this phenomenon: despite an increase in SNO in *Brassica juncea* plants undergoing low temperature stress, Abat and Deswal [15] identified nine proteins undergoing enhanced S-nitrosylation and eight proteins becoming de-nitrosylated. A similar trend was observed following salt stress [51]. Together with our observations, it becomes clear that the accumulation of NO or SNO in plant cells does not concomitantly induce a global response leading to general S-nitrosylation of target proteins. The NO production/turnover and the NO targets for S-nitrosylation may be spatially or temporally separated. The kinetics and the localization of the NO production in leaves as measured by NO-specific fluorophores demonstrated that the NO production begins within the chloroplasts and subsequently propagates into the nucleus and the cytosol [104], [105]. The stress-dependent activation of de-nitrosylases could provide another explanation for de-nitrosylation events in the presence of enhanced NO and nitrite concentrations. In the case of phosphorylation, the phosphorylation status of proteins depends on the activities of kinases and phosphatases. Similarly, the extent of *S*-nitrosylation in any protein will depend on the rates of S-nitrosylation and de-nitrosylation [6]. Recent observations classify GSNO reductase and the thioredoxin/thioredoxin reductase system as denitrosylases [19], [106], [107]. These enzymes might themselves be regulated by NO [108]–[111]. Moreover, de-nitrosylation is not exclusively enzymatically driven. Non-enzymatic de-nitrosylation can occur due to alterations in the cellular redox environment (pH and pO_2_ shifts) [112].

### De-nitrosylation regulates PAL activity in response to acute ozone

S-nitrosylation events can alter the outcome of a signaling pathway by switching on/off target proteins. The regulation of enzyme activity by S-nitrosylation was demonstrated for several plant proteins [10]. Strikingly, S-nitrosylation is often mentioned in conjunction with an inhibition of the enzyme activity, whereas de-nitrosylation can promote the enzyme activity [13]–[15], [28], [113]–[116]. Our quantitative proteomic analysis revealed three enzymes that are involved in different phenolic pathways as being de-nitrosylated in response to ozone fumigation (COMT, PAL2, PPO). PAL is the first and committing enzyme of the phenylpropanoid pathway, which directs the carbon flow from the shikimate pathway to the various branches of the general phenylpropanoid metabolism. The observed increase in the overall PAL activity upon ozone exposure (5-fold) is higher than the increase in enzyme activity that we observed from incubation with SIN, promoting de-nitrosylation (approximately 25%). This difference might be explained by other PTMs, such as de-phosphorylation events, after ozone exposure. Indeed, a phosphorylation site has been identified in the PAL of French bean (*Phaseolus vulgaris* L.). Phosphorylation at this site (Thr545) was associated with a decrease in the V_max_ and an increased turnover of the PAL subunits [78], [117]. Our multiple sequence alignment of the five PAL isoenzymes of *Populus trichocarpa* showed that the phosphorylation site of the French bean PAL corresponds with highly conserved threonine residues in poplar. Analogous to phosphorylation, S-nitrosylation/de-nitrosylation may also modify the catalytic activity of PAL by changing the substrate access to the active center as indicated by the observed increase of the overall *in vitro* PAL activity following incubation with SIN. Future studies with heterologous expressed PAL proteins (i.e. PcPAL2 and PcPAL3) site-directly modified at the Cys557 probably will clarify this initial observation. Interestingly, NO fumigation or NO donor treatment is often associated with a rapid increase in the activity of PAL concomitant with an accumulation of phenolic and flavonoid compounds [118]–[120]. In our work, COMT became significantly de-nitrosylated upon ozone fumigation. COMT is an essential enzyme that is involved in monolignol biosynthesis, influencing the lignin content and lignin structure in poplar [121]. In poplar, the transcription of COMT, and hence the lignin content, can be induced by ozone, leading to an enhanced ozone tolerance due to the antioxidative properties of lignin [122].

As de-nitrosylation of enzymes is often observed with an up-regulation of enzyme activity [19] we hypothesize that de-nitrosylation represents another PTM to regulate enzyme activities, contributing to a fast up-regulation of the metabolic flux through the phenylpropanoid pathway within minutes upon onset of the stress event producing the biosynthetic precursor of all kinds of defense metabolites in ozone-stressed poplars. At longer time scales (hours to days) accumulation of polyphenols would be achieved by an up-regulation of *de-novo* synthesis [44], [74], [82], [123], [124]. This hypothesis, however, needs to be proven by future detailed biochemical analysis of the respective enzymes.

## Supporting Information

Figure S1 **Pictures of** ***Populus*** **x** ***canescens*** **callus tissue.** Leaf (A) and stem (B) explants of poplar were cultured in darkness on callus induction medium for 3 weeks at 20°C. (C) Fully developed calli were transferred to shoot induction medium and maintained under moderate light (16/8 h photoperiod, PPFD 125 µmol photons m^−2 ^s^−1^).
(TIF)

Figure S2 **Experimental setup to test the uptake of ozone into the poplar leaves.** (A) Glass cuvette with enclosed poplar plant. (B) Ozone concentrations measured at the inlet (empty circles) and outlet (filled circles) of the empty cuvette and when the plant was enclosed. (C) Response of poplar assimilation (filled squares) and transpiration rates (empty squares) to acute, short-term ozone exposure. (D) Foliar ozone flux during light and dark periods. Grey shaded box indicates when the light was turned off.
(TIF)

Figure S3 **Western blot showing** ***in vivo*** **S-nitrosylated proteins in callus, leaf tissue (C) and leaves subjected to ozone (O), including controls for false-positives (FP).** Callus and leaf extracts underwent the biotin switch assay, were separated by SDS-PAGE and were blotted onto nitrocellulose membrane. Biotinylated ( = S-nitrosylated) proteins were detected by an anti-biotin antibody. The lower part shows the PonceauS-stained membrane for loading control. The biotin switch assay was repeated three times with similar results.
(TIF)

Figure S4 **Two-dimensional (A) score and (B) scaled and centered loading plots of principal component analysis (PCA) of S-nitrosylated proteins in poplar leaf samples identified using biotin switch assay and LC-MS/MS.** The explained variance (in percentage) and the number of principal components (PC) are reported in x- and y-axes in both (A) and (B) plots. Ellipse in (A) indicates the tolerance based on Hotelling’s T2 with significance level of 0.05. The outer and inner ellipses in (B) indicate 100% and 50% explained variance, respectively. A, control = black circles; ozone-treated = grey triangles; false positive (FP) control = black circle white-filled; FP ozone-treated = grey triangle white-filled; B, each functional group of proteins is indicated with different symbols, zoomed symbols with a dot represent the significantly different proteins between C and O plants tested independently with Student’s t-test (P<0.05 applying a FDR of 5%). Symbol legend: dark red circles = Amino acid metabolism and Protein synthesis, folding and degradation; blue diamonds = Redox and Signaling; cyan triangles-up = Primary metabolism; yellow squares = Secondary metabolism; red triangles-down = Stress; dark green squares = Structural function; grey triangles-down = other; grey squares = not assigned or not identified.
(TIF)

Figure S5 **Multiple alignment of PAL protein sequences from different species.** The alignment was performed with COBALT tool from NCBI. The five isoenzymes of *Populus trichocarpa* PAL (ACC63888.1, EEE89380, ACC63887.1, EEF04645, and XP_002315308) were aligned with PAL1 from parsley (*Petroselinum crispum*: CAA68938.1), Arabidopsis (*Arabidopsis thaliana*: AEC09341.1), tobacco (*Nicotiana tabacum*: BAA22963.1) and pea (*Pisum sativum*: Q01861.1). All of the cysteine residues are highlighted in yellow. Cysteine residue predicted to be targets of S-nitrosylation by GPS-SNO software [76] are highlighted in red. The active center of the PAL is defined by the Ala-Ser-Gly tripeptide (framed in green). Red letters indicate highly conserved positions (identical amino acid in all aligned species) and blue letters indicate less conserved ones.
(DOC)

Table S1 **Full data set for protein identification of (A) S-nitroso-proteins, (B) total proteins from whole cell extracts (WCE) and (C) corresponding protein abundances after label-free quantification and normalization process.** (A) Identified S-nitrosylated proteins of grey poplar callus and leaf tissue (control (C), ozone-fumigated (O)). Only proteins with at least two unique peptides were used for further analyses (black) (grey = proteins with < than 2 unique peptides used for quantification). Enzymes of the polyphenol metabolism are highlighted in bold. (B) Identified total proteins of grey poplar whole cell extracts (WCE) from C and O samples. MASCOT search of acquired spectra conveyed a total of 1,461 detectable proteins in C and O samples with 3 replicates in each. Annotations of proteins are only given for those proteins detected as S-nitrosylated in Table S1A (black). (C) Normalization of protein abundances from S-nitroso-proteins with the averages of the protein abundances from total proteins. The abundance of each S-nitrosylated protein was normalized to the corresponding, averaged abundance in the whole-cell extracts (WCE) of the C and O leaves. For the very low abundant proteins, which were not detected in the WCE, the S-nitroso-protein was normalized to a “calculated total protein” (marked in red). The “calculated total protein” was the S-nitroso-protein abundance multiplied by 100 and divided by the median (i.e. 2.2) of percentages between all S-nitroso-protein and the respective measured total proteins. Log2 fold changes were calculated from the means of the normalized C and O replicates.
(XLSX)

Table S2 **Functional categorization of S-nitrosylated proteins according to MapManBINs (** http://ppdb.tc.cornell.edu/dbsearch/mapman.aspx **).** BINs (major functional categories) and subcategories (subBINs) are given with the corresponding number of proteins.
(DOC)
