# Supplementary figures and images for: S-Nitroso-Proteome in Poplar Leaves in Response to Acute Ozone Stress
Source: PLoS One. 2014 Sep 5;9(9):e106886. doi: 10.1371/journal.pone.0106886 (PMC4156402; doi:10.1371/journal.pone.0106886)

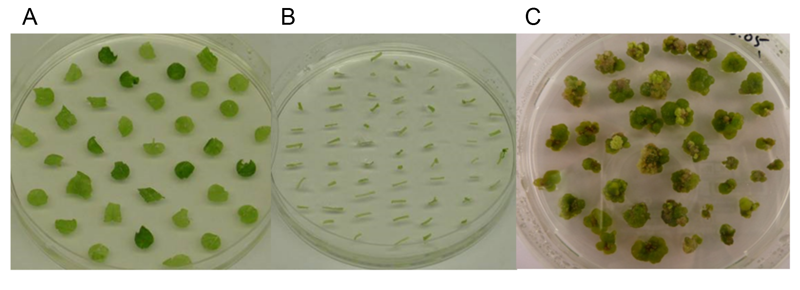

Supplement: Figure S1 — Pictures of Populus x canescens callus tissue. Leaf (A) and stem (B) explants of poplar were cultured in darkness on callus induction medium for 3 weeks at 20°C. (C) Fully developed calli were transferred to shoot induction medium and maintained under moderate light (16/8 h photoperiod, PPFD 125 µmol photons m−2 s−1). (TIF) [file pone.0106886.s001.tif]

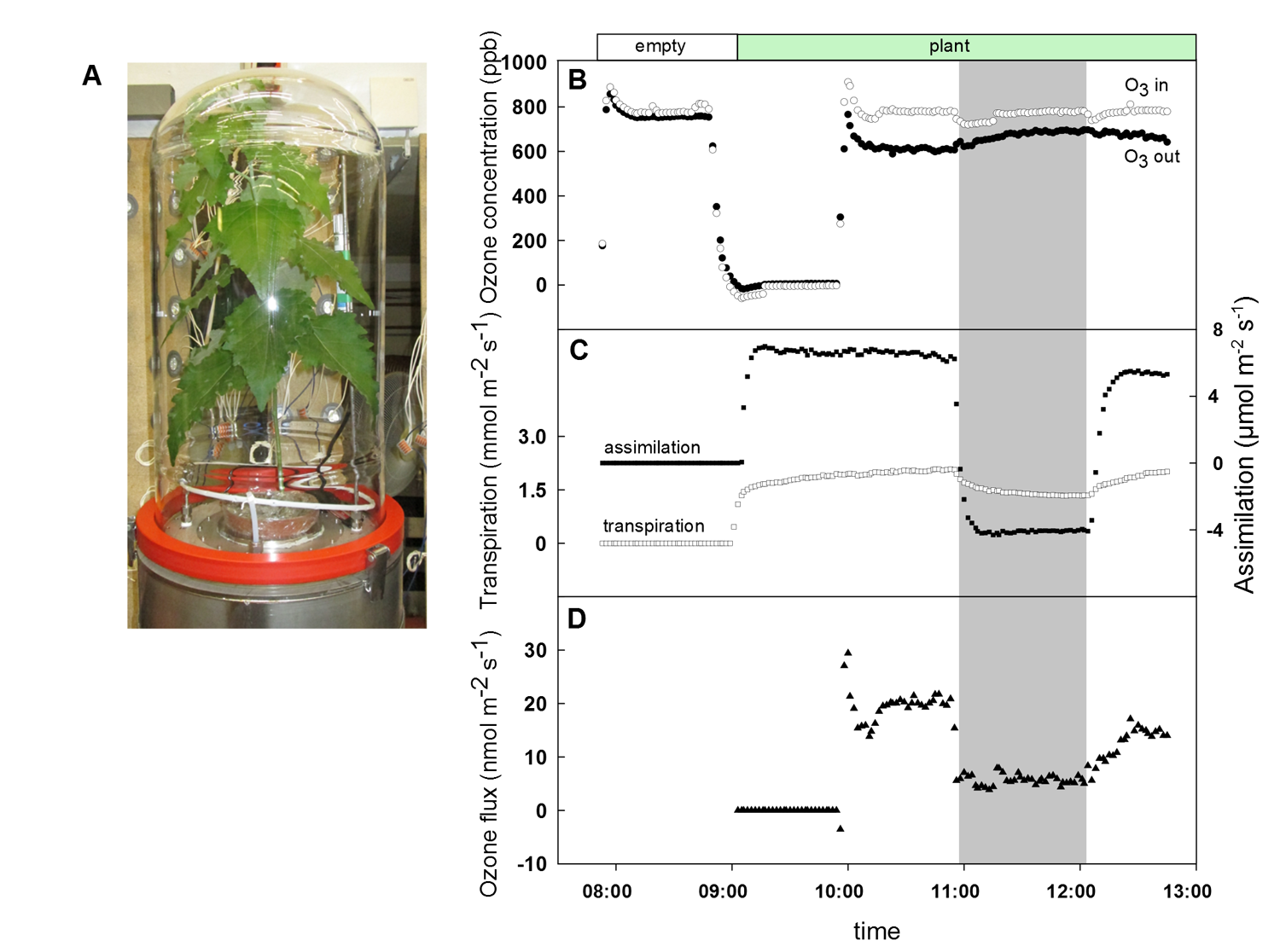

Supplement: Figure S2 — Experimental setup to test the uptake of ozone into the poplar leaves. (A) Glass cuvette with enclosed poplar plant. (B) Ozone concentrations measured at the inlet (empty circles) and outlet (filled circles) of the empty cuvette and when the plant was enclosed. (C) Response of poplar assimilation (filled squares) and transpiration rates (empty squares) to acute, short-term ozone exposure. (D) Foliar ozone flux during light and dark periods. Grey shaded box indicates when the light was turned off. (TIF) [file pone.0106886.s002.tif]

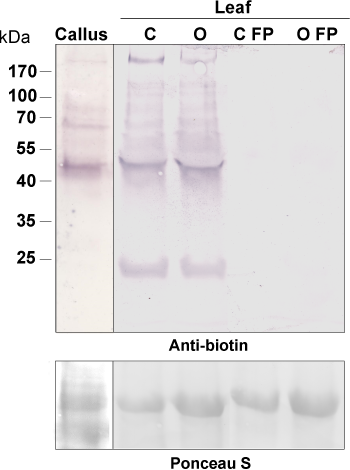

Supplement: Figure S3 — Western blot showing in vivo S-nitrosylated proteins in callus, leaf tissue (C) and leaves subjected to ozone (O), including controls for false-positives (FP). Callus and leaf extracts underwent the biotin switch assay, were separated by SDS-PAGE and were blotted onto nitrocellulose membrane. Biotinylated ( = S-nitrosylated) proteins were detected by an anti-biotin antibody. The lower part shows the PonceauS-stained membrane for loading control. The biotin switch assay was repeated three times with similar results. (TIF) [file pone.0106886.s003.tif]

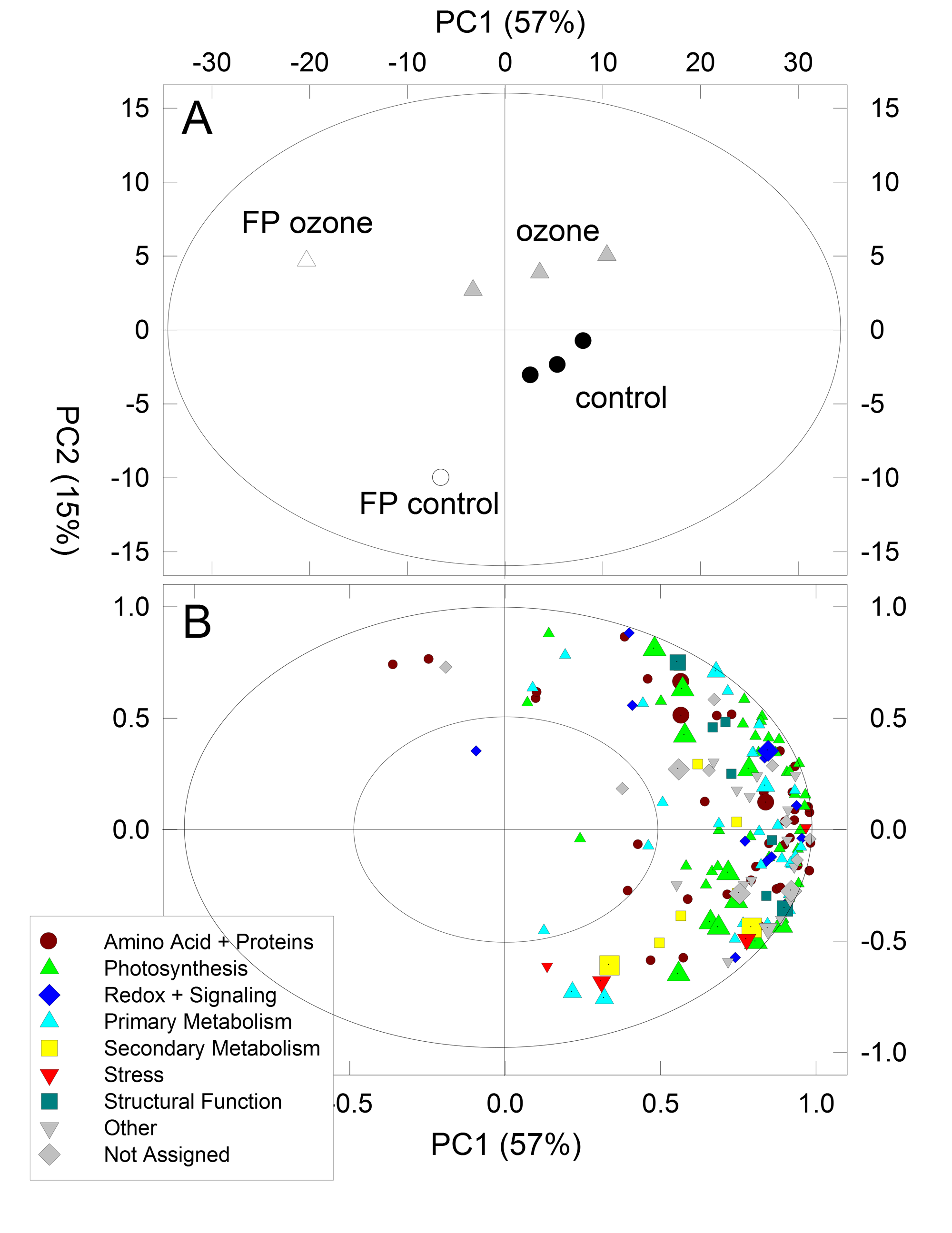

Supplement: Figure S4 — Two-dimensional (A) score and (B) scaled and centered loading plots of principal component analysis (PCA) of S-nitrosylated proteins in poplar leaf samples identified using biotin switch assay and LC-MS/MS. The explained variance (in percentage) and the number of principal components (PC) are reported in x- and y-axes in both (A) and (B) plots. Ellipse in (A) indicates the tolerance based on Hotelling’s T2 with significance level of 0.05. The outer and inner ellipses in (B) indicate 100% and 50% explained variance, respectively. A, control = black circles; ozone-treated = grey triangles; false positive (FP) control = black circle white-filled; FP ozone-treated = grey triangle white-filled; B, each functional group of proteins is indicated with different symbols, zoomed symbols with a dot represent the significantly different proteins between C and O plants tested independently with Student’s t-test (P<0.05 applying a FDR of 5%). Symbol legend: dark red circles = Amino acid metabolism and Protein synthesis, folding and degradation; blue diamonds = Redox and Signaling; cyan triangles-up = Primary metabolism; yellow squares = Secondary metabolism; red triangles-down = Stress; dark green squares = Structural function; grey triangles-down = other; grey squares = not assigned or not identified. (TIF) [file pone.0106886.s004.tif]
