# Supplementary material for: S-Nitroso-Proteome in Poplar Leaves in Response to Acute Ozone Stress
Source: PLoS One. 2014 Sep 5;9(9):e106886. doi: 10.1371/journal.pone.0106886 (PMC4156402; doi:10.1371/journal.pone.0106886)
Supplement: Figure S5 — Multiple alignment of PAL protein sequences from different species. The alignment was performed with COBALT tool from NCBI. The five isoenzymes of Populus trichocarpa PAL (ACC63888.1, EEE89380, ACC63887.1, EEF04645, and XP_002315308) were aligned with PAL1 from parsley (Petroselinum crispum: CAA68938.1), Arabidopsis (Arabidopsis thaliana: AEC09341.1), tobacco (Nicotiana tabacum: BAA22963.1) and pea (Pisum sativum: Q01861.1). All of the cysteine residues are highlighted in yellow. Cysteine residue predicted to be targets of S-nitrosylation by GPS-SNO software [76] are highlighted in red. The active center of the PAL is defined by the Ala-Ser-Gly tripeptide (framed in green). Red letters indicate highly conserved positions (identical amino acid in all aligned species) and blue letters indicate less conserved ones. (DOC) [file pone.0106886.s005.doc]

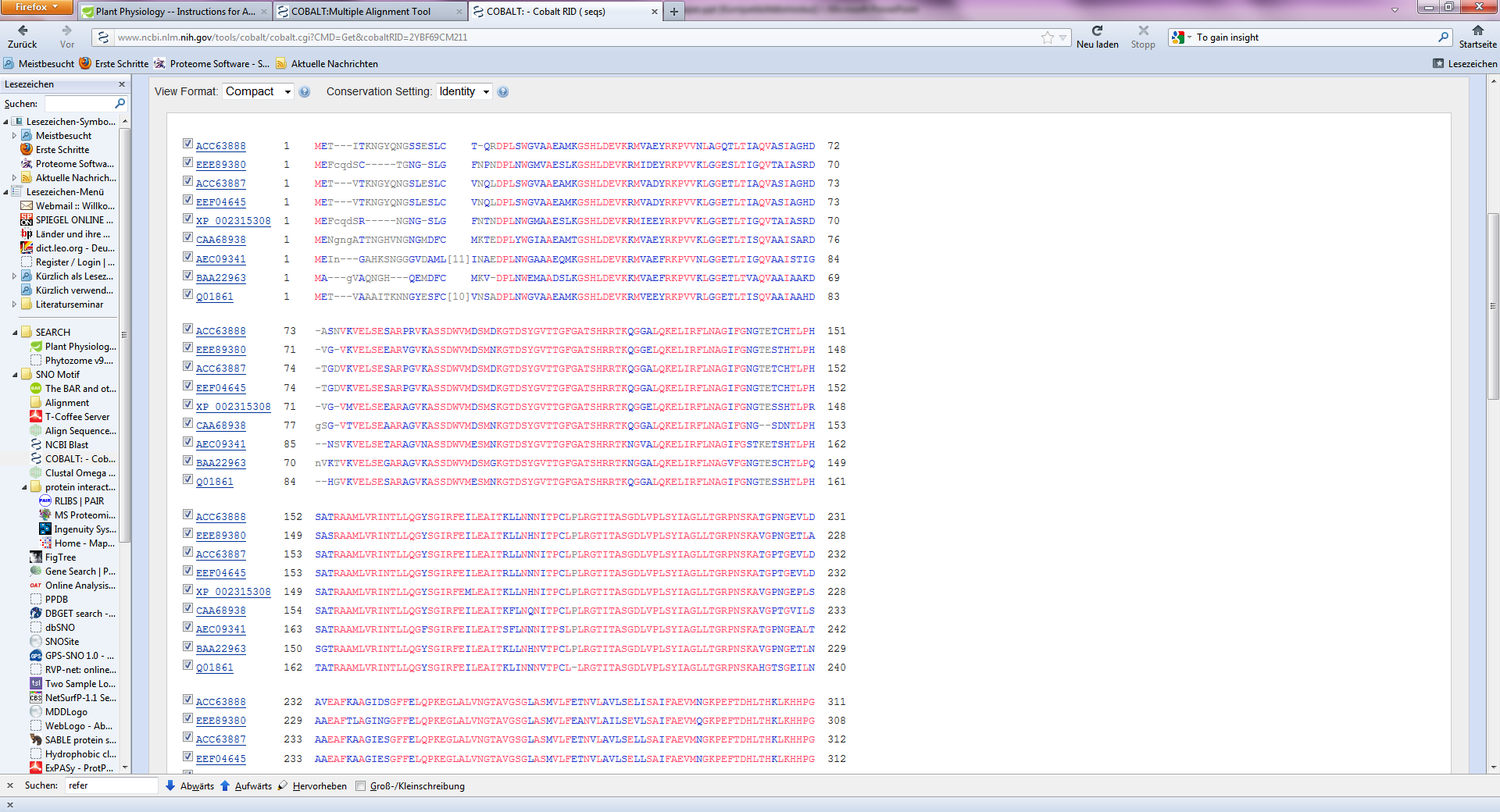


***Populus trichocarpa* PAL1**

***Populus trichocarpa* PAL2**

***Populus trichocarpa* PAL3**

***Populus trichocarpa* PAL4**

***Populus trichocarpa* PAL5**

***Petroselinum crispum* PAL1**

***Arabidopsis thaliana* PAL1**

***Nicotiana tabacum* PAL1**

***Pisum sativum* PAL1**


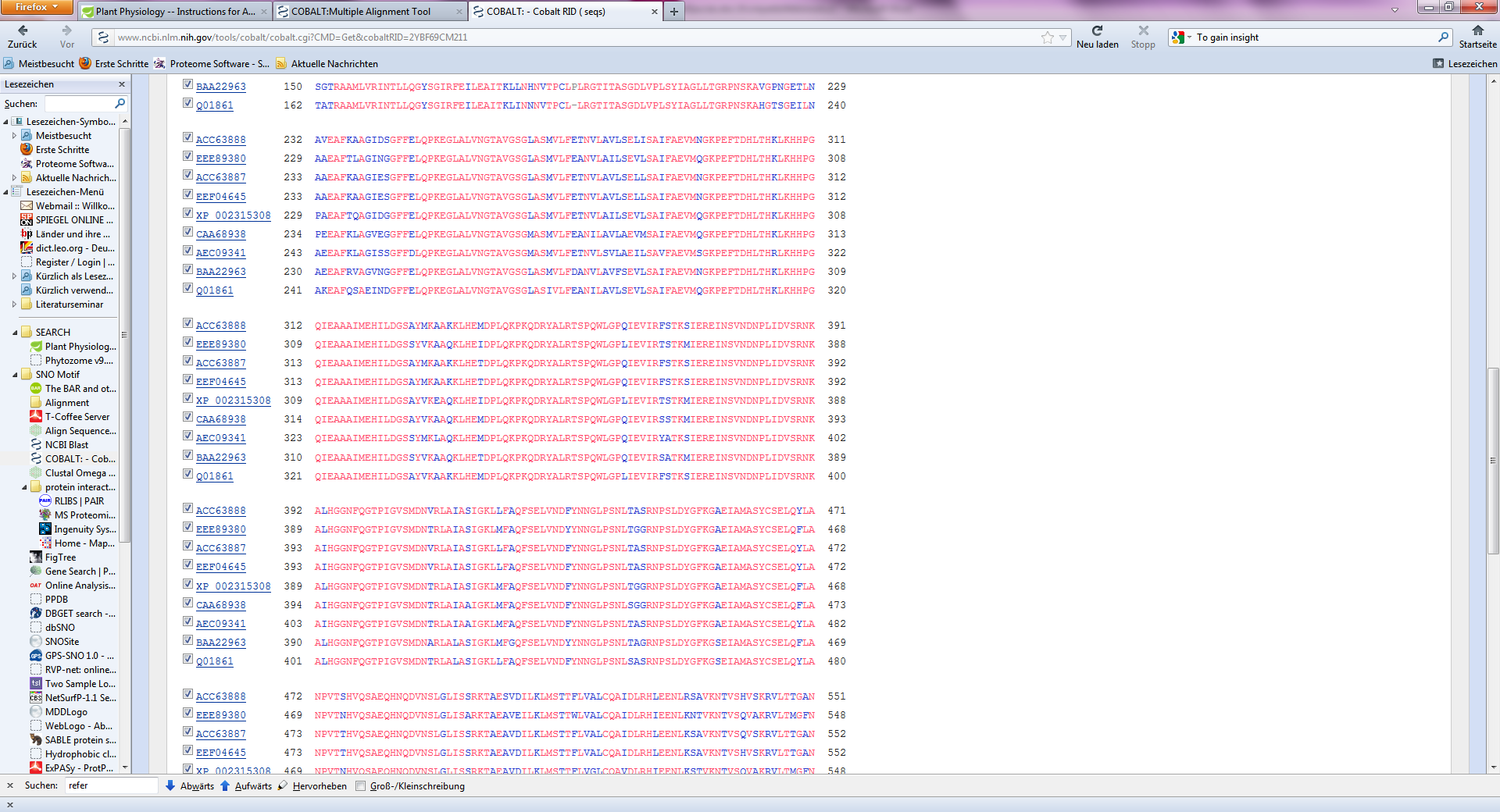


***Populus trichocarpa* PAL1**

***Populus trichocarpa* PAL2**

***Populus trichocarpa* PAL3**

***Populus trichocarpa* PAL4**

***Populus trichocarpa* PAL5**

***Petroselinum crispum* PAL1**

***Arabidopsis thaliana* PAL1**

***Nicotiana tabacum* PAL1**

***Pisum sativum* PAL1**

***Populus trichocarpa* PAL1**

***Populus trichocarpa* PAL2**

***Populus trichocarpa* PAL3**

***Populus trichocarpa* PAL4**

***Populus trichocarpa* PAL5**

***Petroselinum crispum* PAL1**

***Arabidopsis thaliana* PAL1**

***Nicotiana tabacum* PAL1**

***Pisum sativum* PAL1**

***Populus trichocarpa* PAL1**

***Populus trichocarpa* PAL2**

***Populus trichocarpa* PAL3**

***Populus trichocarpa* PAL4**

***Populus trichocarpa* PAL5**

***Petroselinum crispum* PAL1**

***Arabidopsis thaliana* PAL1**

***Nicotiana tabacum* PAL1**

***Pisum sativum* PAL1**

***Populus trichocarpa* PAL1**

***Populus trichocarpa* PAL2**

***Populus trichocarpa* PAL3**

***Populus trichocarpa* PAL4**

***Populus trichocarpa* PAL5**

***Petroselinum crispum* PAL1**

***Arabidopsis thaliana* PAL1**

***Nicotiana tabacum* PAL1**

***Pisum sativum* PAL1**

***Populus trichocarpa* PAL1**

***Populus trichocarpa* PAL2**

***Populus trichocarpa* PAL3**

***Populus trichocarpa* PAL4**

***Populus trichocarpa* PAL5**

***Petroselinum crispum* PAL1**

***Arabidopsis thaliana* PAL1**

***Nicotiana tabacum* PAL1**

***Pisum sativum* PAL1**

**active center**


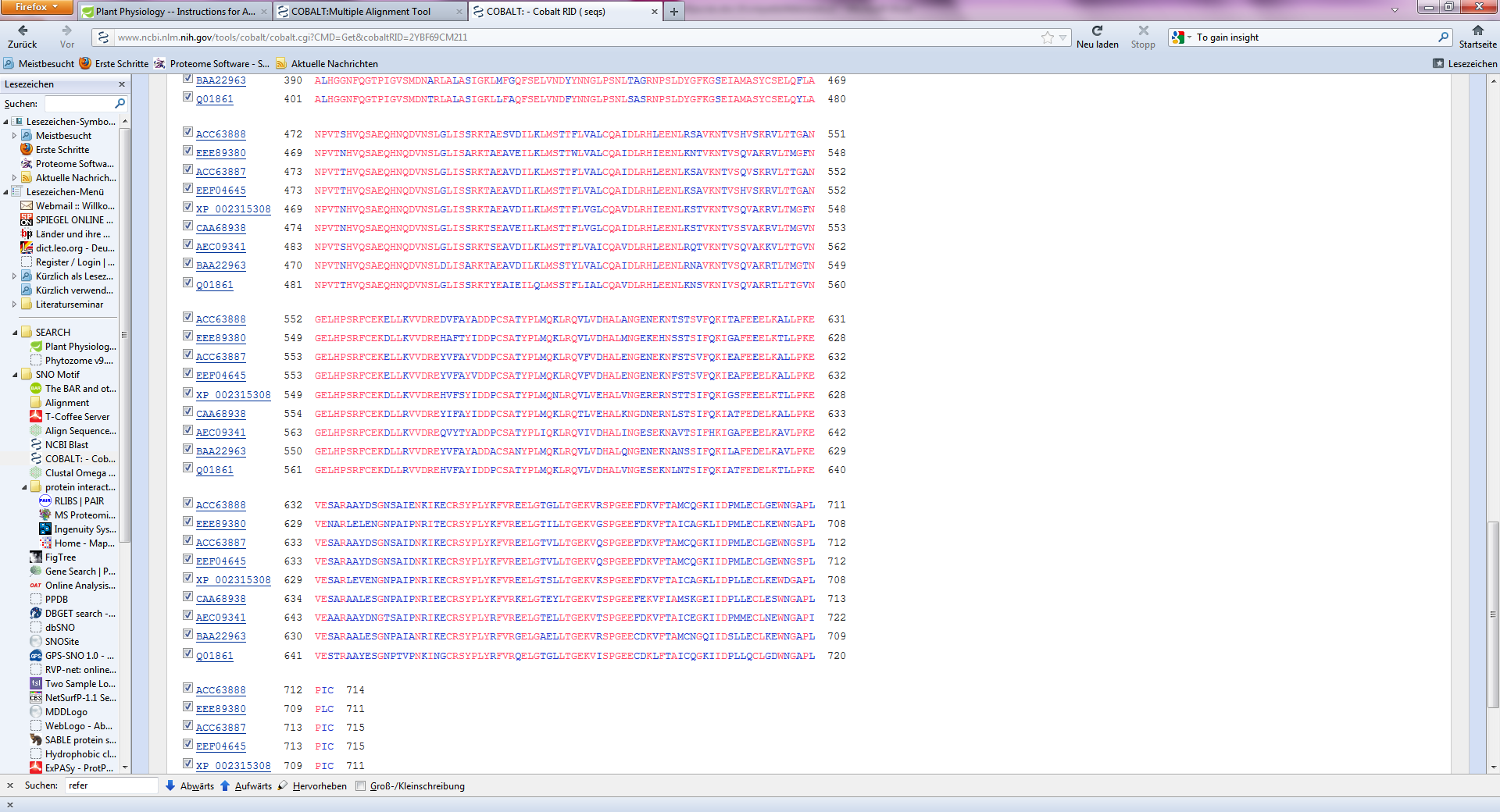


***Populus trichocarpa* PAL1**

***Populus trichocarpa* PAL2**

***Populus trichocarpa* PAL3**

***Populus trichocarpa* PAL4**

***Populus trichocarpa* PAL5**

***Petroselinum crispum* PAL1**

***Arabidopsis thaliana* PAL1**

***Nicotiana tabacum* PAL1**

***Pisum sativum* PAL1**

***Populus trichocarpa* PAL1**

***Populus trichocarpa* PAL2**

***Populus trichocarpa* PAL3**

***Populus trichocarpa* PAL4**

***Populus trichocarpa* PAL5**

***Petroselinum crispum* PAL1**

***Arabidopsis thaliana* PAL1**

***Nicotiana tabacum* PAL1**

***Pisum sativum* PAL1**

**
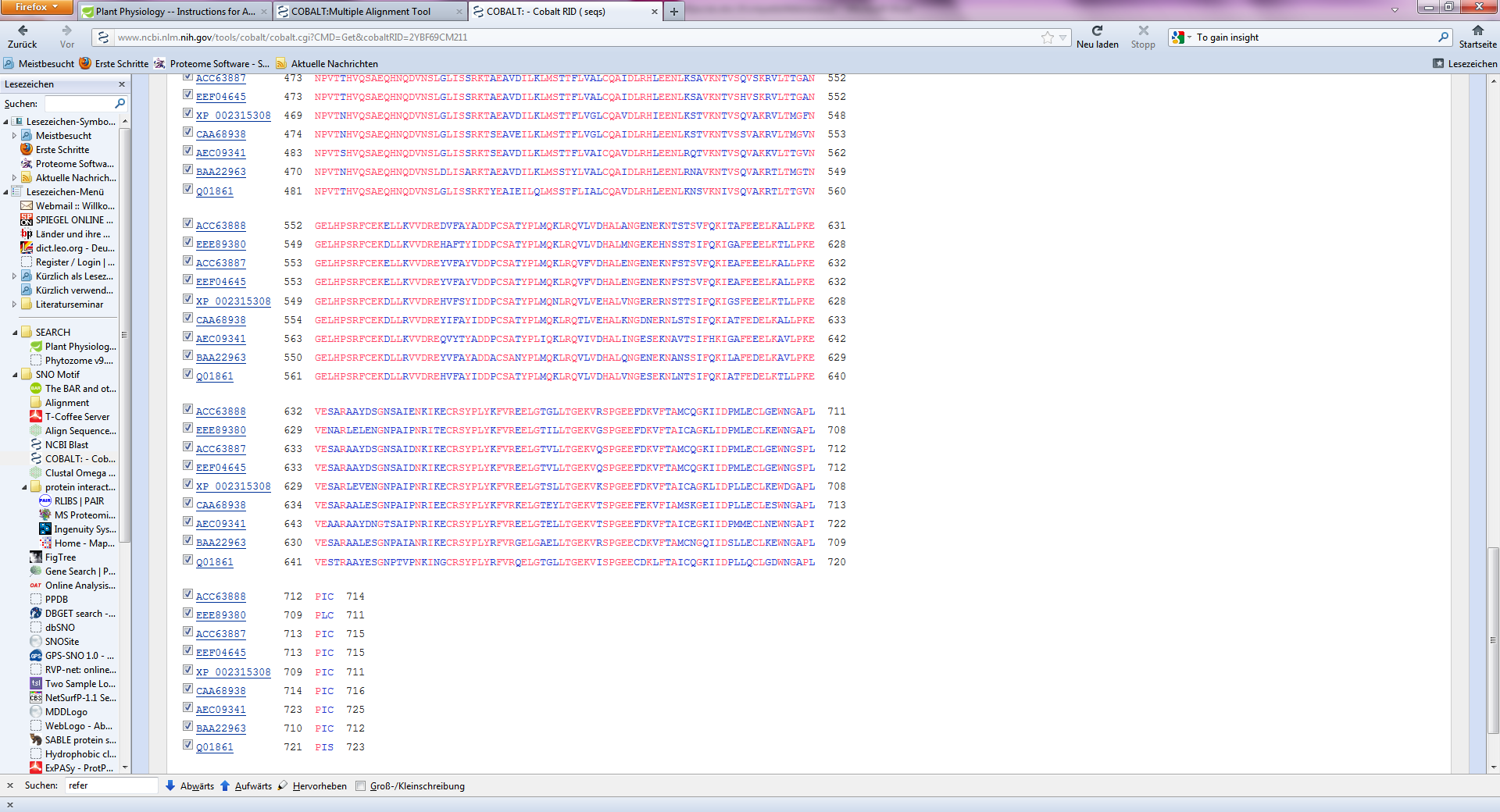
**

***Populus trichocarpa* PAL1**

***Populus trichocarpa* PAL2**

***Populus trichocarpa* PAL3**

***Populus trichocarpa* PAL4**

***Populus trichocarpa* PAL5**

***Petroselinum crispum* PAL1**

***Arabidopsis thaliana* PAL1**

***Nicotiana tabacum* PAL1**

***Pisum sativum* PAL1**

**bv**

***Populus trichocarpa* PAL1**

***Populus trichocarpa* PAL2**

***Populus trichocarpa* PAL3**

***Populus trichocarpa* PAL4**

***Populus trichocarpa* PAL5**

***Petroselinum crispum* PAL1**

***Arabidopsis thaliana* PAL1**

***Nicotiana tabacum* PAL1**

***Pisum sativum* PAL1**

**Figure S5.** Multiple alignment of PAL protein sequences from different species. The alignment was performed with COBALT tool from NCBI. The five isoenzymes of *Populus trichocarpa* PAL (ACC63888.1, EEE89380, ACC63887.1, EEF04645, and XP_002315308) were aligned with PAL1 from parsley (*Petroselinum crispum:* CAA68938.1), Arabidopsis (*Arabidopsis thaliana*: AEC09341.1), tobacco (*Nicotiana tabacum*: BAA22963.1) and pea (*Pisum sativum*: Q01861.1). All of the cysteine residues are highlighted in yellow. Cysteine residue predicted to be targets of S-nitrosylation by GPS-SNO software are highlighted in red. The active center of the PAL is defined by the Ala-Ser-Gly tripeptide (framed in green). Red letters indicate highly conserved positions (identical amino acid in all aligned species) and blue letters indicate less conserved ones.
