# Supplementary material for: S-Nitroso-Proteome in Poplar Leaves in Response to Acute Ozone Stress
Source: PLoS One. 2014 Sep 5;9(9):e106886. doi: 10.1371/journal.pone.0106886 (PMC4156402; doi:10.1371/journal.pone.0106886)
Supplement: Table S2 — Functional categorization of S-nitrosylated proteins according to MapManBINs ( http://ppdb.tc.cornell.edu/dbsearch/mapman.aspx ). BINs (major functional categories) and subcategories (subBINs) are given with the corresponding number of proteins. (DOC) [file pone.0106886.s007.doc]

| **Table S2.** Functional categorization of S-nitrosylated proteins according to MapManBINs (http://ppdb.tc.cornell.edu/dbsearch/mapman.aspx). BINs (major functional categories) and subcategories (subBINs) are given with the corresponding number of proteins. | | | | |
| --- | --- | --- | --- | --- |
| **BIN** | **BIN designation** | **subBIN** | **subBIN designation** | **Number of proteins** |
| **29** | **protein** |  |  | **26** |
|  |  | 29.2 | protein.synthesis | 7 |
|  |  | 29.3 | protein.targeting | 2 |
|  |  | 29.5 | protein.degradation | 8 |
|  |  | 29.6 | protein.folding | 7 |
|  |  | 29.8 | protein.assembly and cofactor ligation | 2 |
| **1** | **photosynthesis** |  |  | **35** |
|  |  | 1.1 | PS lightreaction | 17 |
|  |  | 1.2 | PS photorespiration | 4 |
|  |  | 1.3 | PS calvin cycle | 14 |
| **13** | **amino acid metabolism** |  |  | **13** |
|  |  | 13.1 | amino acid metabolism.synthesis | 9 |
|  |  | 13.2 | amino acid metabolism.degradation | 4 |
| **26** | **misc** |  |  | **11** |
|  |  | 26.13 | misc.acid and other phosphatases | 1 |
|  |  | 26.23 | misc.rhodanese | 1 |
|  |  | 26.28 | misc.GDSL-motif lipase | 2 |
|  |  | 26.3 | misc.gluco-, galacto- and mannosidases | 3 |
|  |  | 26.3 | misc.other Ferredoxins and Rieske domain | 1 |
|  |  | 26.31 | misc.fibrillins | 1 |
|  |  | 26.7 | misc.oxidases - copper, flavone etc. | 1 |
|  |  | 26.9 | misc.glutathione S transferases | 1 |
| **35** | **not assigned** |  |  | **11** |
| **21** | **redox** |  |  | **10** |
|  |  | 21.1 | redox.thioredoxin | 1 |
|  |  | 21.2 | redox.ascorbate and glutathion | 4 |
|  |  | 21.5 | redox.periredoxins | 3 |
|  |  | 21.6 | redox.dismutases and catalases | 2 |
| **4** | **glycolysis** |  |  | **10** |
|  |  | 4.1 | glycolysis.UGPase | 1 |
|  |  | 4.12 | glycolysis.enolase | 1 |
|  |  | 4.13 | glycolysis.PK | 2 |
|  |  | 4.5 | glycolysis.pyrophosphate-fructose-6-P phosphotransferase | 1 |
|  |  | 4.7 | glycolysis.aldolase | 2 |
|  |  | 4.8 | glycolysis.TPI | 1 |
|  |  | 4.9 | glycolysis.glyceraldehyde 3-phosphate dehydrogenase | 2 |
| **20** | **stress** |  |  | **6** |
|  |  | 20.1 | stress.biotic | 3 |
|  |  | 20.2 | stress.abiotic | 3 |
| **16** | **secondary metabolism** |  |  | **5** |
|  |  | 16.1 | secondary metabolism.isoprenoids | 1 |
|  |  | 16.2 | secondary metabolism.phenylpropanoids | 2 |
|  |  | 16.8 | secondary metabolism.flavonoids | 2 |
| **8** | **TCA / org.transformation** |  |  | **5** |
|  |  | 8.1 | TCA / org.transformation.TCA | 2 |
|  |  | 8.2 | TCA / org.transformation.other organic acid transformaitons | 2 |
|  |  | 8.3 | TCA / org.transformation.carbonic anhydrases | 1 |
| **10** | **cell wall** |  |  | **4** |
| **19** | **tetrapyrrole synthesis** |  |  | **4** |
| **31** | **cell** |  |  | **4** |
| **2** | **major CHO metabolism** |  |  | **3** |
| **30** | **signalling** |  |  | **3** |
| **11** | **lipid metabolism** |  |  | **2** |
| **12** | **N-metabolism** |  |  | **2** |
| **18** | **Co-factor and vitamine metabolism** |  |  | **2** |
| **27** | **RNA** |  |  | **2** |
| **14** | **S-assimilation** |  |  | **1** |
| **15** | **metal handling** |  |  | **1** |
| **23** | **nucleotide metabolism** |  |  | **1** |
| **25** | **C1-metabolism** |  |  | **1** |
| **5** | **fermentation** |  |  | **1** |
| **7** | **OPP** |  |  | **1** |
| **9** | **mitochondrial electron transport / ATP synthesis** |  |  | **1** |
